# Förster Resonance Energy Transfer Nanoplatform Based on Recognition-Induced Fusion/Fission of DNA Mixed Micelles for Nucleic Acid Sensing

**DOI:** 10.1021/acsnano.1c00156

**Published:** 2021-05-07

**Authors:** Setareh Vafaei, Francia Allabush, Seyed R. Tabaei, Louise Male, Timothy R. Dafforn, James H. R. Tucker, Paula M. Mendes

**Affiliations:** † School of Chemical Engineering, University of Birmingham, Edgbaston, Birmingham B15 2TT, United Kingdom; ‡ School of Chemistry, University of Birmingham, Edgbaston, Birmingham B15 2TT, United Kingdom; § School of Biosciences, University of Birmingham, Edgbaston, Birmingham B15 2TT, United Kingdom

**Keywords:** FRET, micelle, signal enhancement, DNA sensing, lipid oligonucleotide conjugates

## Abstract

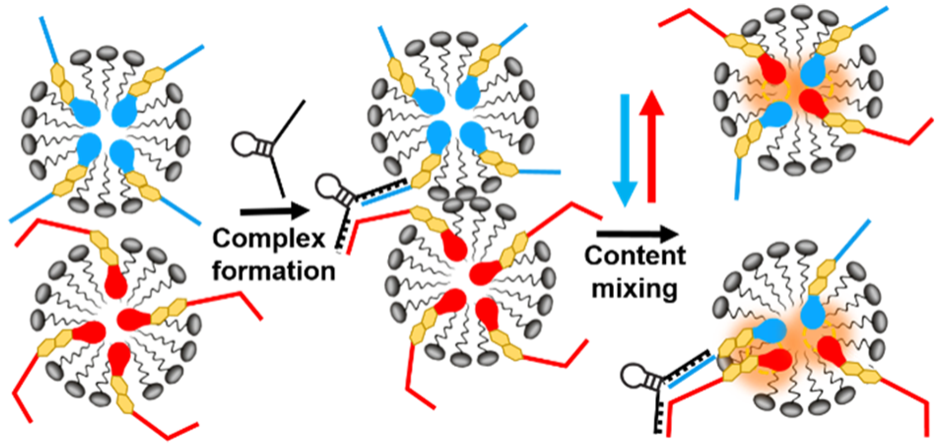

The dynamic nature of micellar nanostructures is employed to form a self-assembled Förster resonance energy transfer (FRET) nanoplatform for enhanced sensing of DNA. The platform consists of lipid oligonucleotide FRET probes incorporated into micellar scaffolds, where single recognition events result in fusion and fission of DNA mixed micelles, triggering the fluorescence response of multiple rather than a single FRET pair. In comparison to conventional FRET substrates where a single donor interacts with a single acceptor, the micellar multiplex FRET system showed ∼20- and ∼3-fold enhancements in the limit of detection and FRET efficiency, respectively. This supramolecular signal amplification approach could potentially be used to improve FRET-based diagnostic assays of nucleic acid and non-DNA based targets.

## Introduction

The growing demand for application of less time and cost-consuming solutions in clinical diagnostics necessitates increasing the sensitivity and efficiency of biological assays beyond the limits of optical biosensing. Förster resonance energy transfer (FRET) is one of the most popular methods for measuring distance between molecules at the nanoscale, making it useful for probing molecular binding/unbinding events. While many FRET-based biosensors have been developed for a wide range of analyte–receptor recognition events, these systems are inherently limited in their sensitivity due to the limited brightness of organic fluorescent dyes. This shortcoming has stimulated the application of a range of nanomaterials as FRET-based sensing platforms, including plasmonic nanoantennas,^1,2^ dye-loaded inorganic^3^ and polymeric^4^ nanoparticles (NPs), quantum dots,^5^ and dendrimeric nanostructures.^6^ However, these approaches require laborious chemical synthesis and demanding fabrication steps. Moreover, the outcome strongly depends on the optimization of several parameters including the surface chemistry, size and shape of the nanomaterials, and the position and orientation of the fluorophore relative to the nanomaterial surface.^7−9^

In a typical FRET assay, one binding event results in the close spatial proximity and subsequent energy transfer between a donor and an acceptor fluorophore. One way to achieve label amplification in FRET is to design a platform in which one recognition event brings several rather than single FRET pairs into close proximity.

To enhance the number of FRET pair engagements per recognition event, we propose a strategy involving co-solubilization of amphiphilic FRET probes in micellar nanoparticles. Micelles are thermodynamically stable supramolecular structures that have the capacity to accommodate hydrophobic as well as amphiphilic agents. Micelles are also dynamic structures, exhibiting a range of processes such as chain exchange while in equilibrium and splitting (fission) and re-formation (fusion) during re-equilibration initiated by external stimuli. Importantly, they spontaneously self-assemble into tunable monodisperse nanoparticles. Utilizing the dynamic properties of micellar structures, in the present work, we set about designing a FRET nanoplatform for DNA sensing.

DNA detection is vital in biological research and medical diagnostics.^10^ For DNA assays, signal and target amplification is crucial for detection at the ultralow concentrations found in clinical samples. Typically, this is achieved by target amplification, which entails enzymatic multiplication of DNA fragments using the polymerase chain reaction (PCR). However, this technique is hindered by large costs, high complexity, sequence bias, and sensitivity to contamination.^11^ For this reason, attention has been diverted to developing target amplification-free methods of DNA detection.^12^

Here, we demonstrate label signal amplification using lipid oligonucleotide mixed micelles in a FRET-based sandwich DNA hybridization assay. Lipid oligonucleotide conjugates (LOCs) are synthetically derived amphiphilic molecules comprised of short strands of DNA or RNA connected to lipophilic moieties that are typically steroidal or hydrocarbon chain based.^13^ The exclusive properties of LOCs make them appealing as building blocks for supramolecular applications and biosensing.^14−16^

**Figure 1:**
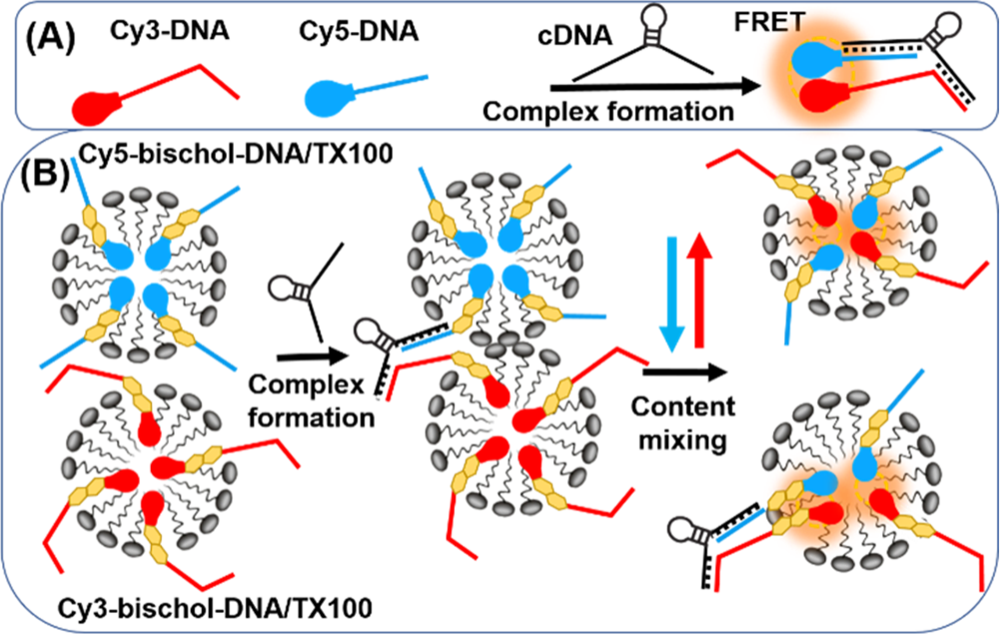
Signal amplification in a FRET-based sandwich DNA hybridization assay. (A) Conventional FRET platform where a single binding event results in the close proximity of only one FRET pair. (B) Mixed micelle FRET system. A single binding event results in the close proximity of multiple FRET pairs due to micelle content mixing.

In our approach, to build the FRET platform, lipid oligonucleotide conjugates with DNA on one end and FRET pairs Cy3 or Cy5 on the opposite end are co-solubilized with the non-ionic surfactant Triton X-100 (TX100) to produce mixed micelles. Addition of complementary DNA (cDNA) brings the two micelle populations, Cy3-bischol-DNA (donor micelle) and Cy5-bischol-DNA (acceptor micelle), into close contact (Figure 1). This promotes micelle content mixing and results in FRET. The signal amplification in this system stems from the fact that one binding event brings several FRET pairs incorporated in each micelle into close proximity. An ∼20-fold enhancement in the sensitivity in comparison to a conventional FRET substrate of the same composition was obtained.

## Results and Discussion

**Scheme 1:**
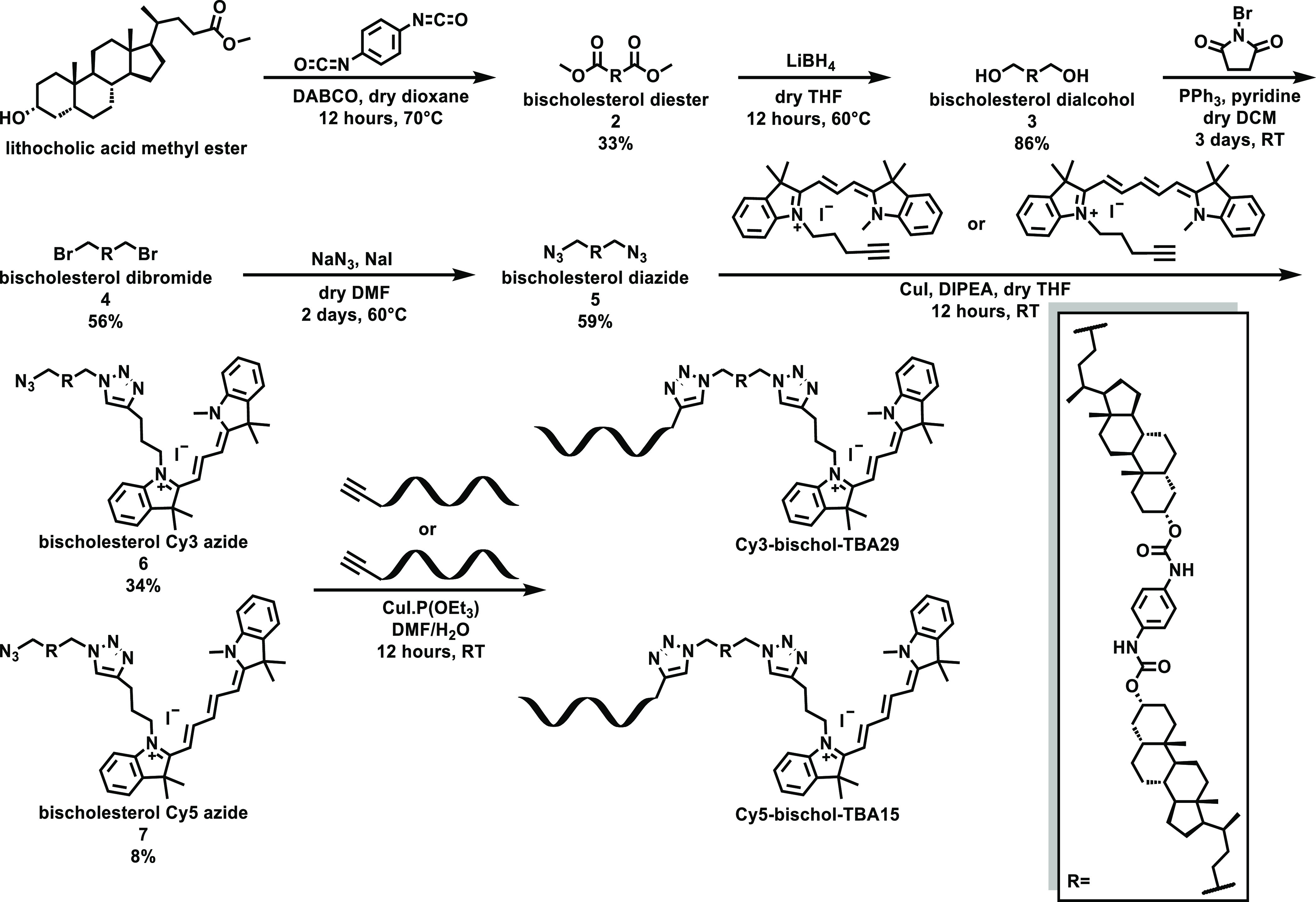
Synthesis of the Fluorescent LOC Probes

The design of our LOC probes consisted of an oligonucleotide coupled to a fluorescent bivalent steroidal system, as depicted in Scheme 1. Two conjugated steroidal units were selected over one to ensure stable micelle formation. The use of one cholesterol unit was considered to be insufficient for self-assembly of the amphiphilic probes into stable micelles.^17^

Probe synthesis was initiated by the creation of the bivalent steroidal system. Two molecules of lithocholic acid were coupled to both sides of *p*-phenylene diisocyanate to generate the lipid section (bischolesterol diester **2**). Prior to this coupling, lithocholic acid was esterified to prevent reaction of the acid with the isocyanate groups on the phenyl ring. Functional groups on both sides of the bivalent lipid were then altered to allow attachment of either DNA or dyes to the ends of the molecule. The ester groups were first hydrolyzed to alcohols in good yield using lithium borohydride. The resulting dialcohol (**3**) was then converted to the dibromide (**4**) in acceptable yields by reacting with triphenylphosphine and *N*-bromosuccinimide. The last functional group conversion entailed reaction of the dibromide (**4**) with sodium azide to generate the diazide (**5**). The diester (**2**), dibromide (**4**), and diazide (**5**) molecules were highly crystalline products, with crystal structures obtained (see Supporting Information Figures S9–S11). Cy3 and Cy5 dyes were selected for their chemical versatility, hydrophobicity, and well-studied use in FRET experiments.^18^ Alkyne modified versions of the dyes were synthesized following a literature procedure^18^ and coupled to one end of the diazide lipid system *via* copper catalyzed azide–alkyne cycloaddition chemistry (Scheme 1, compounds **6** and **7**). Finally, short, single strands of 5′-alkyne modified DNA were appended to the other end of the molecule by copper catalyzed azide–alkyne cycloaddition to complete probe formation. Thrombin binding aptamer sequences TBA15^19^ and TBA29^20^ were chosen for potential sensing of non-DNA based targets. However, the experiments in this work are mainly focused on sensing DNA. The synthesized probes were purified by reversed-phase HPLC and characterized by mass spectrometry (ESI). Two different fluorescent LOC probes were utilized in sensing experiments, Cy3-bischol-TBA29 (Cy3-bischol-DNA) and Cy5-bischol-TBA15 (Cy5-bischol-DNA), along with corresponding control compounds without the bischolesterol units Cy3-TBA29 (Cy3-DNA) and Cy5-TBA15 (Cy5-DNA). All the sequences are listed in the Supporting Information Table S5.

**Figure 2:**
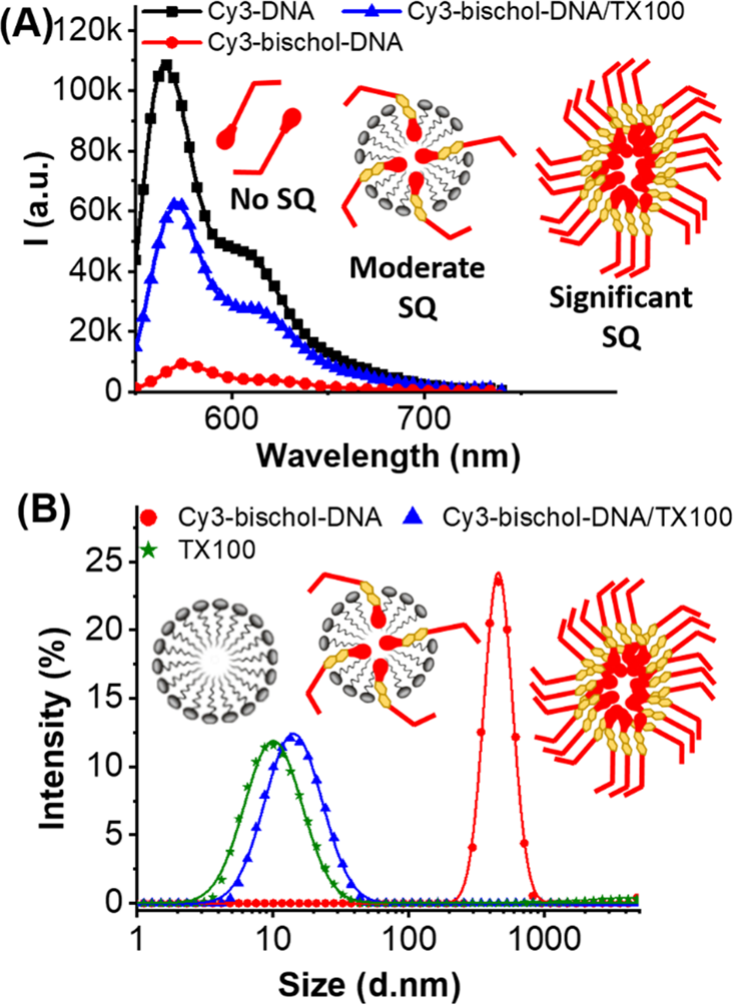
(A) Representative fluorescence emission spectra of Cy3-DNA (black squares), Cy3-bischol-DNA/TX100 (blue triangles), and Cy3-bischol-DNA (red circles) excited at 522 nm. Concentrations were kept equal at 0.125 μM. Schematic illustrations of Cy3-bischol-DNA aggregates, Cy3-bischol-DNA/TX100 mixed micelles, and Cy3-DNA monomers inferred from the level of self-quenching (SQ) are also shown. (B) DLS data indicating the average size distribution of Cy3-bischol-DNA (red circles), Cy3-bischol-DNA/TX100 (blue triangles), and TX100 micelles (green stars). “*k*” in the values refers to (×1000) for the arbitrary units.

To prepare the self-assembled DNA–mixed micelle nanostructures, dye-bischol-DNA was incubated with the non-ionic surfactant TX100. Figure 2A compares the fluorescence emission spectra of Cy3-DNA, Cy3-bischol-DNA, and Cy3-bischol-DNA/TX100 at the same concentration (0.125 μM). The fluorescence intensity at the peak wavelength for Cy3-bischol-DNA was approximately 12-fold lower than that of Cy3-DNA. This observation indicates significant self-quenching of the dye in Cy3-bischol-DNA samples, suggesting that the Cy3-bischol-DNA molecules form highly packed aggregates due to the hydrophobicity of the bischolesterol moiety. This contrasts with the Cy3-DNA sample, with its high fluorescence intensity indicating a minimal intermolecular interaction at this concentration (Figure 2A).

Notably, the fluorescence intensity of the Cy3-bischol-DNA/TX100 sample was ∼7-fold higher than that of Cy3-bischol-DNA, indicating a much lower degree of self-quenching. As expected, an increase in the fluorescence intensity upon addition of TX100 was also observed in the corresponding Cy5-containing samples (Figure S26). This observation suggests that the addition of TX100 disrupts the Cy3-bischol-DNA aggregates, resulting in the formation of mixed micelles with fewer Cy3 molecules in the micelle core (Figure 2A). Nevertheless, the mixed micelle system still displays considerable self-quenching, suggesting the presence of a relatively high local concentration of the Cy3 molecules in the core of the mixed micelles.

To further verify the disruption of Cy3-bischol-DNA aggregates by TX100, the sizes of Cy3-bischol-DNA and Cy3-bischol-DNA/TX100 samples were measured by dynamic light scattering (DLS; Figure 2B). The average size of Cy3-bischol-DNA aggregates in the Tris buffer was ∼286.6 ± 59.9 nm. After addition of TX100, the size was significantly reduced to ∼13.0 ± 1.1 nm, indicating disruption of the aggregates and formation of mixed micelles. As expected, the size of the mixed micelle particles was slightly higher than that of the TX100 micelles (10.8 ± 0.5 nm), as the DNA strands protrude out of the micelles, resulting in an increase in the hydrodynamic size of the particles. Notably, the fact that the sizes of the surfactant alone and the mixed system are so similar suggests that a spherical micelle is indeed forming.

To gain more insight into the aggregation state of the Cy3-bischol-DNA sample and the mechanism underlying the fluorescence reduction, we performed fluorescence polarization (FP) measurements. This experiment exploits the fact that when identical fluorophores in close proximity are excited with polarized light, the emitted light gets depolarized through a so-called homo-FRET process. This energy transfer between fluorophores of the same kind occurs in clusters of identical fluorophores when the emission spectrum of a fluorophore overlaps with its own absorption spectrum.^21,22^ As shown in Figure S27, the emission and absorption spectra of Cy3 overlap significantly. The FP of Cy3-bischol-DNA samples (FP = 0.044 ± 0.02) was significantly lower than that of Cy3-bischol-DNA/TX100 (FP = 0.27875 ± 0.004), indicating strong homo-FRET in the highly packed aggregates of randomly oriented Cy3-bischol-DNA molecules. FP is also sensitive to the rotational mobility and hence partly to the size of aggregates; fluorophores associated with larger aggregates rotate at a lower rate in the course of their excited-state lifetime. Therefore, fluorophores associated with larger aggregates are expected to show higher polarization compared to the fluorophores associated with smaller aggregates. Given that the size of Cy3-bischol-DNA (286.6 ± 59.87 nm) is over 20-fold of that of Cy3-bischol-DNA/TX100 micelles (13.03 ± 1.14 nm), the size effect cannot account for the very low FP observed in the Cy3-bischol-DNA sample compared to the Cy3-bischol-DNA/TX100 micelles sample.

The FRET activities of dye-DNA and dye-bischol-DNA/TX100 mixed micelle systems were next examined using a DNA target strand (cDNA) complementary to both TBA15 and TBA29. The two probes assemble in the presence of the complementary strand (Figure S28), bringing Cy3 and Cy5 FRET pairs close together to produce a FRET signal (enhanced acceptor (*i.e.*, Cy5) emission upon donor (*i.e.*, Cy3) excitation).

**Figure 3:**
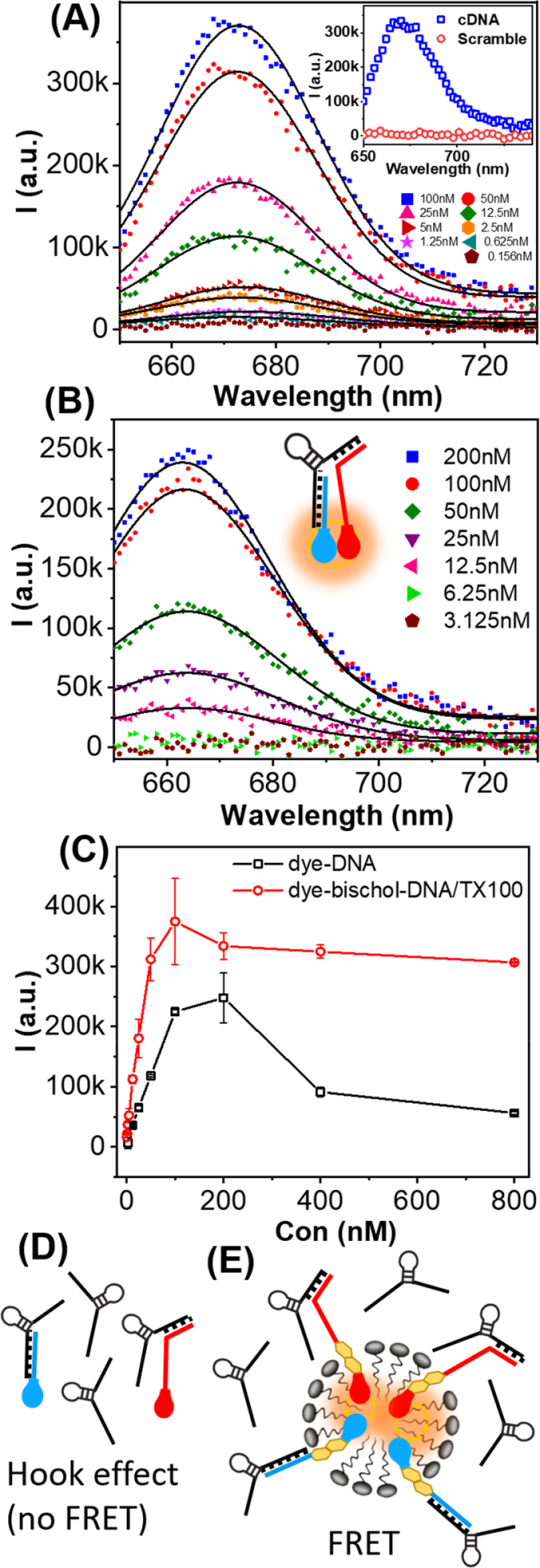
Background corrected Cy5 emission upon Cy3 excitation (522 nm) at different cDNA concentrations for (A) dye-bischol-DNA/TX100 and (B) dye-DNA. The inset in panel A compares cDNA with scrambled DNA at 100 nM. (C) Cy5 emission peak intensity as a function of cDNA concentration for dye-DNA (black squares) and dye-bischol-DNA/TX100 (red circles). (D) Schematic illustration of the “hook effect” in the dye-DNA system. Oversaturation of probes prevents FRET. (E) Schematic illustration of FRET in the presence of excess cDNA in the dye-bischol-DNA/TX100 system showing no hook effect despite oversaturation of FRET probes. “*k*” in the values refers to (×1000) for the arbitrary units.

Figure 3A shows Cy5 emission spectra (FRET signal) of the dye-bischol-DNA/TX100 mixed micelle system at a series of different cDNA concentrations (0.156–100 nM). Addition of increasing amounts of cDNA up to a concentration of 100 nM resulted in an associated increase in the Cy5 fluorescence intensity upon excitation of Cy3, proving that FRET occurs efficiently in the mixed micelle system. In this system, the FRET signal was discernible at cDNA concentrations as low as 0.625 nM.

To show that the FRET signal resulted from specific cDNA recognition, a scrambled DNA sequence was tested as a control (Table S5). Negligible FRET was observed even at high concentrations of scrambled DNA (100 nM; Figure 3A, inset). In addition, no FRET was observed upon incubation with DNA strands complementary only to either the Cy5-bischol-DNA or the Cy3-bischol-DNA (Figure S29). Further control experiments were performed by replacing Na^+^ with Mg^2+^ in the buffer. The presence of Mg^2+^ results in folding of the DNA probes used in this study (*i.e.*, TBA G-quadruplex), which prevents binding with cDNA.^23^ As expected, FRET was significantly decreased in the presence of Mg^2+^ (Figure S30).

Figure 3B shows FRET signals for the dye-DNA system under the same experimental conditions with concentrations similar to the mixed micelle system. Notably, the FRET signal was not discernible when cDNA was at or below a concentration of 6 nM. Hence, the limit of detection (LoD) was much higher for the dye-DNA system (12.5 nM) than for the dye-bischol-DNA/TX100 mixed micelle system (0.625 nM). Therefore, a >20-fold increase in the FRET sensitivity was achieved with the mixed micelle system in comparison to the conventional design approach (dye-DNA).

To gain further insight into the origin of the observed enhancement, we compared the spectral characteristics of the dyes in the dye-DNA and the dye-bischol-DNA/TX100 systems (Figure S31). We determined Förster radii of 6.61 and 6.76 nm for the dye-DNA and the dye-bischol-DNA/TX100 assemblies, respectively (Supporting Information Table S6). Notably, while the Förster radius of the dye-bischol-DNA/TX100 was only slightly greater than that of the dye-DNA, the mixed micelle system showed a significant increase in the FRET signal. Moreover, while the emission peaks of the dyes are slightly red-shifted in the micelle system, the shapes of the spectra of the dyes in the two systems are very similar (Figure S32). These observations suggest that the increased FRET signal in the mixed micelle system stems from differences in the FRET construct and signaling events rather than the spectral properties of the fluorophores themselves.

Indeed, this marked enhancement in sensitivity verifies that one binding event brings several rather than single FRET pairs together (Figure 1B). The reason for this is that each cDNA interacts with two different aptamer-based probes in separate micelles (one with Cy3-bischol-DNA, the other with Cy5-bischol-DNA). Once the (Cy3-bischol-DNA)-cDNA-(Cy5-bischol-DNA) complex is formed, two different mixed micelle populations are brought into close contact, facilitating the mixing of micelle contents (Cy3-bischol-DNA and Cy5-bischol-DNA). Similar behavior has been reported in liposomes where hybridization of a membrane-anchored DNA strand forced bilayers into proximity and triggered liposome fusion.^24−26^ Importantly, we observed that the equilibrium size of the micelles after addition of the cDNA did not change significantly (DLS results shown in Figure S33), indicating that micelles did not form larger stable aggregates after cDNA-triggered micellar clustering. This suggests that content mixing most likely occurs through a fusion−fission (merging–splitting) process, where donor and acceptor micelles are transiently merged upon cDNA sandwich hybridization and, consequently, DNA probes can exchange and then break into freshly formed smaller micelles (Figure 1B). In an attempt to verify the ability of cDNA to bring dye-bischol-DNA/TX100 micelles into close proximity, we performed gel electrophoresis analysis (2% agarose, Figure S34). Two bands were observed in the presence of 100 nM target cDNA (lane 3), while only one band was observed in the absence of target cDNA (lane 2) or in the presence of scrambled DNA sequence (lane 4). Therefore, the low mobility band which was observed only in the presence of the cDNA (lane 3) indicates the presence of micelle clusters that are formed upon cDNA sandwich hybridization.

The occurrence of these processes at equilibrium has been extensively studied experimentally and theoretically.^27,28^ An alternative explanation is that a bound DNA probe exits the full micelle to the aqueous phase and then enters the other micelle. However, this mechanism is unlikely due to the strong hydrophobicity of the bischolesterol moiety in the DNA probes.

It is important to note that spontaneous content mixing before target binding was insignificant in the time scale of our experiment (30 min; Figure S35), which can be explained by the long-range Coulombic repulsions between micelles caused by the presence of the negatively charged DNA probes. Consequently, content mixing and hence FRET only occur after addition of cDNA (Figure 3A, inset), where hybridization with the DNA probes overcomes the repulsion, bringing the micelles into physical contact and enforcing content mixing. Due to the small size of the mixed micelles (∼13 nm), this process brings several Cy3 and Cy5 molecules in the cores of the micelles into a distance range for FRET (1–10 nm). Notably, this enhanced FRET signal is not produced solely by complex formation between FRET pairs but by content mixing mediated by a single binding event.

To further characterize the two systems, we conducted experiments at cDNA concentrations at or higher than that of the FRET pairs (≥125 nM). Figure 3C shows the FRET peak (*i.e.*, emission peak intensity of Cy5 upon excitation of Cy3) as a function of cDNA concentration in the two systems. In the dye-DNA system, the FRET signal increased linearly with the cDNA concentration when below the concentration of the donor (≤200 nM), showing characteristics of a single donor–acceptor FRET interaction. However, higher concentrations of cDNA resulted in a significant decrease in the FRET signal. This observed decrease is due to a phenomenon termed the “hook effect”, an effect which is commonly reported in sandwich assays.^29^ The hook effect occurs when an excess of the analyte (cDNA) results in simultaneous oversaturation of donors and acceptors (Cy3-DNA and Cy5-DNA). This results in more 1:1 (probe:target) complexes, thereby inhibiting their association in desired 1:2 complexes and attenuating the FRET signal (Figure 3D).

In the mixed micelle sensing system, although the FRET signal also reached a maximum (at a target concentration of 100 nM), in contrast to the dye-DNA system, the signal did not decrease significantly at higher cDNA concentrations (Figure 3C). Assuming that both donors and acceptors are oversaturated at cDNA concentrations several fold higher than that of DNA probes (*i.e.*, 800 nM; Figure 3E), this observation suggests that the close proximity of FRET pairs in the core micelle after content mixing is sufficient for FRET in the presence of excess cDNA, where 1:1 complexes might predominate over sandwich complexes. Taken together, this result indicates that both cDNA–FRET pair complex formation and resulting micelle content mixing are responsible for multiple signaling events, producing enhanced FRET in the mixed micelle system. These results further suggest that the dynamic interfaces of micelles significantly enhance molecular recognition efficiency when compared to molecularly dispersed solutions. Such potential of the interfaces of nanomaterials for improved sensing has been utilized before in a wide range of recognition systems.^30,31^

**Figure 4:**
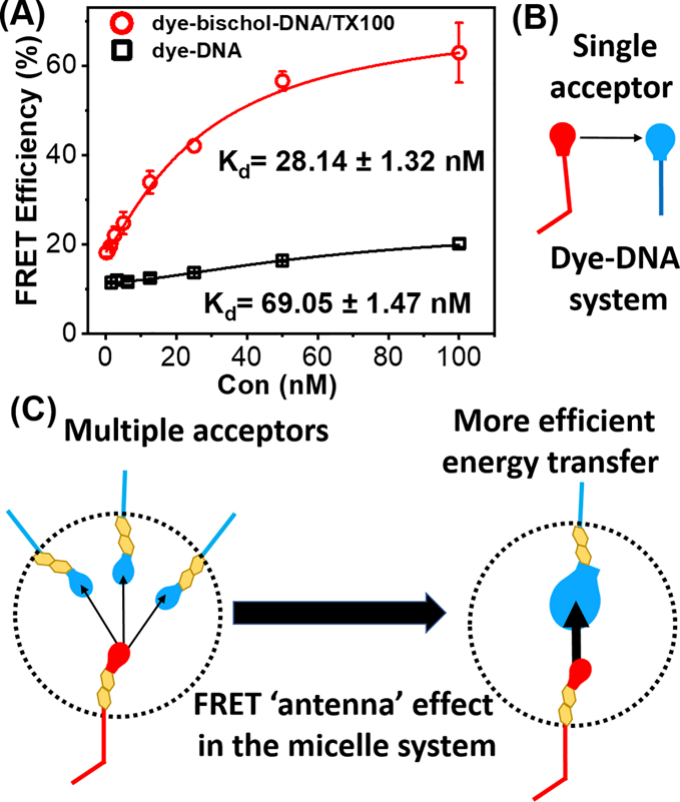
(A) FRET efficiency (*I*_Cy5_/(*I*_Cy5_ + *I*_Cy3_)) of dye-bischol-DNA/TX100 mixed micelles and dye-DNA as a function of cDNA concentration. (B) Schematic illustration of FRET in the dye-DNA system where one donor interacts with one acceptor. (C) Schematic illustration of the FRET antenna effect in the dye-bischol-DNA/TX100 mixed micelles, where multiple FRET interactions result in the enhancement of the FRET efficiency.

To gain further insight into the mechanism underlying the improvement in the limit of detection in the mixed micelle system, we compared the FRET efficiency of the two systems as a function of cDNA concentration (Figure 4A). A, ∼3-fold increase in the FRET efficiency was observed at all cDNA concentrations in the mixed micelle system. Since identical FRET pairs were used in the two systems, the observed difference in the FRET efficiency is expected to stem from the difference in the FRET constructs rather than the nature of the dyes.

Since thrombin binding aptamers were utilized in this study, we also tested the FRET signal generation upon incubation with thrombin. Thrombin was detected in both dye-DNA and dye-bischol-DNA/TX100 systems. However, similar to the case of DNA detection, the FRET efficiency was higher in the mixed micelle system (Figure S36), indicating that amplified sensing through observed FRET enhancement is not limited to DNA detection.

In the dye-DNA sensing system, FRET occurs between single donors and acceptors (Figure 4B). However, in the mixed micelle sensing system, FRET involves interactions between multiple FRET pairs (Figure 4C). Utilizing multiple rather than single FRET pairs, in the FRET constructs, has been shown to be an effective strategy to obtain significant FRET enhancement.^32^ In particular, in biological systems where multiple structures can interact simultaneously, multiplexed FRET has been shown to facilitate detection of interactions at distances greater than 100 Å using common organic fluorophores.^33^ FRET enhancement through multiplexed FRET is shown to mainly be achieved by a mechanism termed the “antenna” effect. This is where the probability of FRET events rise with the increasing number of acceptors per donor, without a change in the donor–acceptor distance.^34^ In theory, for *n* identical acceptors located at the same distance from a single donor, the distance at which energy transfer is 50% efficient (*i.e.*, the Förster radius) increases by a factor of *n*^(1/6)^.^35^ Consequently, the same value of FRET efficiency as in the single donor–single acceptor system case can be achieved at longer distances in the multiple acceptor system. Therefore, by increasing the number of acceptors engaged in each recognition event in the micellar platform, the Förster radius and the value of FRET efficiency can be increased.

Among other physical parameters, FRET efficiency also depends on the relative orientation of the donor and acceptor dipoles. In a multiplexed system, the increase in the number of randomly distributed acceptors increases the probability of a favorable relative dipole orientation for the donor, enhancing the FRET efficiency. This effect is called the “FRET surplus”,^36^ which, in addition to the antenna effect, further enhances the FRET efficiency in a multiplexed system. Considering the relative abundance of acceptors per donor, confined within the nanosized (∼13 nm) mixed micelles (Figure S33), FRET enhancement due to the antenna and surplus effect is plausible (Figure 4C).

In addition, an ∼2.5-fold decrease in the apparent *K*_d_ was observed in the mixed micelle system (Figure 4A). Because identical recognition elements were used in the two systems, the observed difference in the apparent *K*_d_ is expected to originate from differences between solution and surface hybridization,^37^ rather than the affinity of DNA probes for the cDNA. The observed increase in the affinity could be explained by the relatively higher local concentration of the probed DNAs at the micelle interface in comparison to that of the solution phase, which increases the probability of stable hybridization between target cDNA and probe DNAs in states not possible under solution conditions.^38^

Moreover, the content mixing of the micelles mediated by initial recognition events brings both DNA probes into the same nanosized micelles and hence facilitates subsequent sandwich hybridizations between DNA probes and target cDNA.

## Conclusions

In summary, we have developed a signal amplification strategy for FRET sensing of DNA, taking advantage of (i) the dynamic nature of micellar structures and (ii) efficiency enhancement in multiplexed FRET constructs. The approach relies on the content mixing of micelles containing fluorescent lipid oligonucleotide conjugates (LOCs), mediated by recognition of target DNA. In comparison to the conventional single donor–single acceptor design, the limits of detection and FRET efficiency in the mixed micelle system were enhanced by factors of ∼20 and ∼3, respectively.

Nucleic acid detection based approaches have become a rapid and reliable technology for viral detection,^39^ and the recent COVID-19 pandemic demands improvement of such assays for better epidemic prevention and control.^40^ The signal amplification approach developed in this work can potentially be used to improve FRET-based diagnosis of viruses such as SARS-CoV-2.

Finally, as shown by our detection of thrombin, this signal enhancement strategy can be used for detection of non-DNA based targets (*e.g.*, proteins), using different molecular recognition elements and a range of hydrophobic anchors (*e.g.*, lipid-conjugated antibodies).^41^

## Experimental Section

### Procedure for Micellar Solution Preparation

Cy3-bischol-DNA and Cy5-bischol-DNA were separately incubated in buffer solutions containing 10 mM Tris, 150 mM NaCl, and 0.04% (w/v) TX100 (pH 7.4) for 30 min at room temperature. The two were then combined, cDNA was added, and the resulting solution (100 μL) was incubated at room temperature for 30 min. The final concentration of Cy3-bischol-DNA and Cy5-bischol-DNA were 125 and 200 nM, respectively. cDNA concentration was varied from 0 to 800 nM. Dye-DNA solutions were prepared in identical conditions, with the omission of TX100.

### Dynamic Light Scattering Experiments

The average sizes of Cy3-bischol-DNA and bischol-DNA in 10 mM Tris +150 mM NaCl with and without the presence of TX100 were measured by DLS using a Malvern Zetasizer Nano ZS (Malvern Instruments Nordic AB, MAL1040112, Greve, Denmark). The device was equipped with a 633 nm He–Ne laser and operated at an angle of 173°. All measurements were performed in a solvent-resistant microcuvette (ZEN0040, Malvern, Germany) with a sample volume of 100 μL at 25 °C. The average diameter for each particle was obtained from five measurements. Data analysis was performed using Malvern’s Zetasizer software. Since the excitation wavelength of Cy5 is close to the DLS instrument laser wavelength, bischol-DNA was used instead of Cy5-bischol-DNA.

### Fluorescence Experiments

The fluorescence measurements were performed using a CLARIOstar microplate reader (BMG Labtech, Germany). Samples were excited at 522 nm (Cy3 excitation), and emission spectra were collected between 550 and 650 nm for single dye Cy3 experiments and 550–740 nm for two dye FRET experiments. The FRET efficiency was calculated according to the following equation: *E* = *I*_Cy5_/(*I*_Cy5_ + *I*_Cy3_), where *I*_Cy5_ and *I*_Cy3_ are the peak fluorescence intensities of the acceptor and donor, respectively.

### Fluorescence Polarization Experiments

Fluorescence polarization measurements were performed on a PerkinElmer LS-50B spectrophotometer (Beaconsfield, England). The instrument was equipped with a Xenon discharge lamp (half height < 10 μs, 60 Hz) and a red-sensitive R928 photomultiplier. The excitation and emission were set to 522 and 570 nm, respectively, at room temperature. Both excitation and emission slits with a band pass of 10 nm were used for all of the measurements. The polarization was calculated using the following equation:
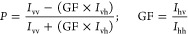
where *I*_vv_ is the intensity with the polarizers vertical and vertical (excitation and emission), *I*_vh_ is the intensity with the polarizers vertical and horizontal (excitation and emission), and GF is the instrumental correction factor. GF is calculated by measuring the polarized components of fluorescence of the probe with horizontally polarized excitation. All of the measurements were analyzed using the FL WinLab software.

### Agarose Gel Electrophoresis Experiments

The gel electro-mobility shift assay was performed on a 2% native agarose gel with 1× TBE buffer using 1× TBE buffer as a running buffer. A sucrose loading buffer was prepared by dissolving 4 g in 10 mL MilliQ water. Five μL of this buffer was then added to 25 μL of each sample. Twenty-five μL aliquots of the resulting solutions containing Triton-X (0.04%), Cy3-bischol-TBA29 (125 nM), Cy5-bischol-TBA15 (200 nM), and complementary DNA (100 nM) or scrambled DNA (100 nM) was then loaded into wells, and the gel was run at 130 V for 1 h. After electrophoresis, the gel was stained with GelRed nucleic acid gel stain (Biotium) and visualized under UV transillumination with a ChemiDoc gel imager from Bio-Rad.

### UV Absorption Experiments

The UV absorption measurements for Cy3 and Cy5 containing samples were conducted using an UV–vis–near-IR spectrophotometer (Varian Cary-5000) in a 10 mm optical path quartz cuvette.

## Supporting Information Available

Supplementary figures and characterization data; additional details about the synthesis and characterization of lipid oligonucleotide conjugates; spectral properties of FRET pairs Förster radius estimation (PDF)
