# Supplementary material for: Förster Resonance Energy Transfer Nanoplatform Based on Recognition-Induced Fusion/Fission of DNA Mixed Micelles for Nucleic Acid Sensing
Source: ACS Nano. 2021 May 7;15(5):8517–24. doi: 10.1021/acsnano.1c00156 (PMC8158853; doi:10.1021/acsnano.1c00156)
Supplement: Supplementary file 1 — nn1c00156_si_001.pdf [file nn1c00156_si_001.pdf]

Förster Resonance Energy Transfer Nanoplatform Based on Recognition-Induced Fusion/Fission of DNA Mixed-Micelles for Nucleic Acid Sensing

Setareh Vafaei, [†,‡] Francia Allabush,[†,‡,§] Seyed R. Tabaei,[†] Louise Male,[§] Timothy R. Daffron,[⊥] James H. R. Tucker, [*,§] Paula M. Mendes[*,†]

[†] School of Chemical Engineering, University of Birmingham, Edgbaston, Birmingham, B15 2TT, UK

[§] School of Chemistry, University of Birmingham, Edgbaston, Birmingham, B15 2TT, UK

[⊥] School of Biosciences, University of Birmingham, Edgbaston, Birmingham, B15 2TT, UK

Table of Contents

General materials and methods 3

Synthesis 3

Synthesis of the bivalent lipid system 4

Synthesis of bischolesterol diester 2 5

Synthesis of bischolesterol dialcohol 3 7

Synthesis of bischolesterol dibromide 4 9

Synthesis of bischolesterol diazide 5 11

Crystal structure determination of bischolesterol diester 2, bischolesterol dibromide 4, and bischolesterol diazide 5 13

Crystal data for bischolesterol diester 2 14

Crystal data for bischolesterol dibromide 4 14

Crystal data for bischolesterol diazide 5 15

General procedure for the synthesis of bischolesterol dye conjugates 16

HPLC purification of the bischolesterol dye conjugates 17

Synthesis of bischolesterol Cy3 azide 6 17

Synthesis of bischolesterol Cy5 azide 7 19

Synthesis and purification of oligonucleotides 22

Standard synthesis of complementary, half-complementary, scrambled, and alkyne modified strands 22
Ultramild synthesis of Cy3 and Cy5 modified control strands 22
Purification of complementary, half-complementary, scrambled, and alkyne modified strands 23
Purification of Cy3 and Cy5 modified control strands 24
Oligonucleotide characterisation 24
Synthesis of fluorescent LOC probes 24
Purification of fluorescent LOC probes 25
Fluorescent LOC probe characterisation 25
Oligonucleotide and fluorescent LOC probe data 26
Additional Data 37
References 43

General materials and methods

Reagents and solvents were purchased from commercial suppliers and used without further purification, unless otherwise stated. Column chromatography was carried out using open columns packed with Merck grade 60 silica gel topped with 0.5cm of sand. TLC analysis was performed on Merck silica gel 60 silica sheets. ^{1}H and ^{13}C NMR spectra were obtained on a Bruker AVIII400 spectrometer. Chemical shifts (δ) are given in ppm and are relative to the residual solvent peak. Electrospray mass (ESI-MS) spectra were measured by either Waters micromass LCT electrospray time-of-flight (ES-TOF), Waters Xevo G2-XS, or Synapt G2S mass spectrometers. IR spectroscopy was performed on dry samples with a Varian 660-IR FT-IR spectrometer. A Stuart SMP10 melting point apparatus was used to determine melting points of compounds in open glass capillary tubes. Preparative/semi preparative HPLC purification and analytical HPLC were performed on Agilent Technologies 1260 Infinity systems. Milli-Q water purified with a Millipore Elix-Gradient A10 system (resistivity > 18μΩ.cm, TOC ≤ 5ppb, Millipore, France) was used for DNA sample preparation and in DNA containing experiments. Fluorescence data was obtained using a microplate reader (BMG Labtech, Germany). DLS experiments were performed on a Malvern Zetasizer Nano ZS (Malvern Instruments Nordic AB, MAL1040112, Greve, Denmark).

Synthesis

Lithocholic acid methyl ester, Cy5 monoalkyne, and Cy3 monoalkyne were synthesised following established procedures.[1, 2]

Synthesis of the bivalent lipid system

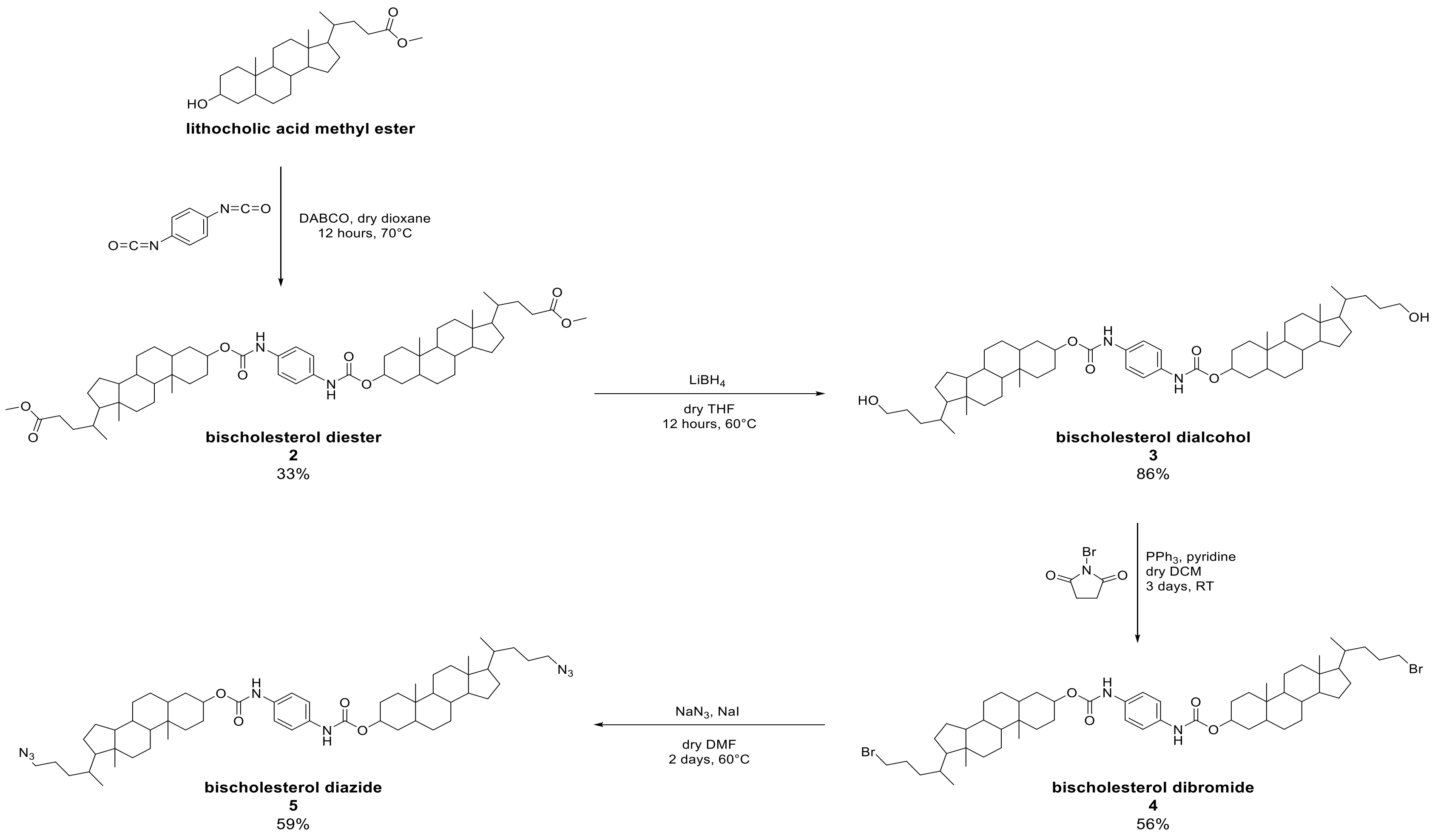

Scheme S1. Synthesis of the bivalent lipid system.

Synthesis of bischolesterol diester 2

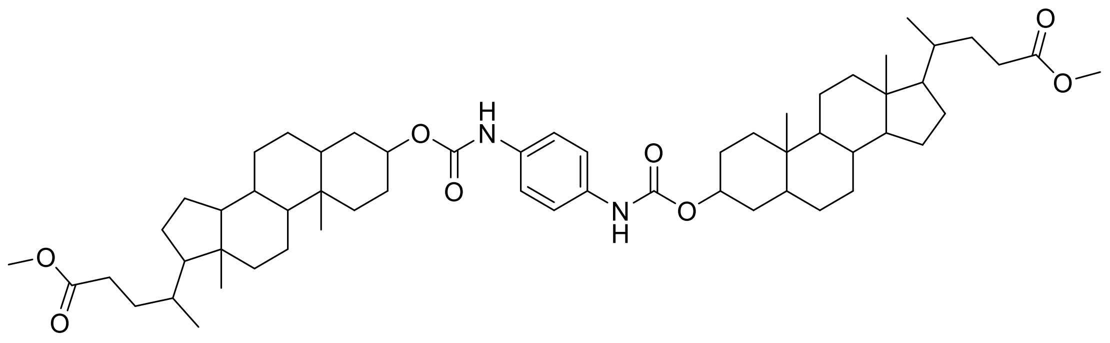

Adapted from Kobuke[3]

Phenylene diisocyanate (205mg, 1.28mmol) and 1,4-diazabicyclo[2.2.2]octane (DABCO, 30mg, 0.26mmol) were added to a solution of lithocholic acid methyl ether (1g, 2.56mmol) in dry dioxane (20ml) under argon at room temperature. The solution was heated to 70°C and left to stir under argon at this temperature overnight. The reaction was then cooled to RT, and the solvent evaporated under reduced pressure. Aqueous 1N HCl was then added to the white solid and the resulting suspension was extracted with DCM until the organic phase was clear. The organic phases were combined, dried over magnesium sulphate, and evaporated under vacuum. The crude product was washed with methanol, dissolved in a small volume of hot chloroform, and purified by flash chromatography with an eluent of 3:1 diethyl ether:hexane. The appropriate fractions were combined and evaporated to afford a white solid (394mg, 33%).

^{1}H NMR (400MHz, $CDCl_3$) δ 7.30 (s, 4H), 6.41 (s, 2H), 4.76-4.68 (m, 2H), 3.67 (s, 6H), 2.39-0.92 (m, 75H), 0.67 (s, 6H). ^{13}C NMR (100MHz, $CDCl_3$) δ 174.8 (C=O), 153.6 (C=O), 134.0 (Ar-C), 119.9 (Ar-CH), 75.7 (CH), 56.9 (CH), 56.4 (CH), 51.5 (OCH_3), 43.1 (C), 42.3 (CH), 40.9 (CH), 40.5 (CH_2), 36.1 (CH), 35.6 (CH), 35.4 (CH_2), 34.9 (C), 32.9 (CH_2), 31.4 (CH_2), 31.3 (CH_2), 28.4 (CH_2), 27.3 (CH_2), 27.2 (CH_2), 26.6 (CH_2), 24.4 (CH_2), 23.5 (CH_3), 21.1 (CH_2), 18.5 (CH_3), 12.3 (CH_3). HRMS (TOF-ESI +ve) (*m/z*): $[M+Na]^+$ calcd for $C_{58}H_{88}N_2O_8Na$, 963.6438; found 963.6440. IR neat (cm^{-1}): 3361 (m, NH, carbamate), 2938 (s, CH, aliphatic), 2864 (s, CH, aliphatic), 1718 (s, C=O, ester/carbamate), 1548 (s, C=C, aromatic), 1522 (s, C=C, aromatic). Melting point: 184-185°C.

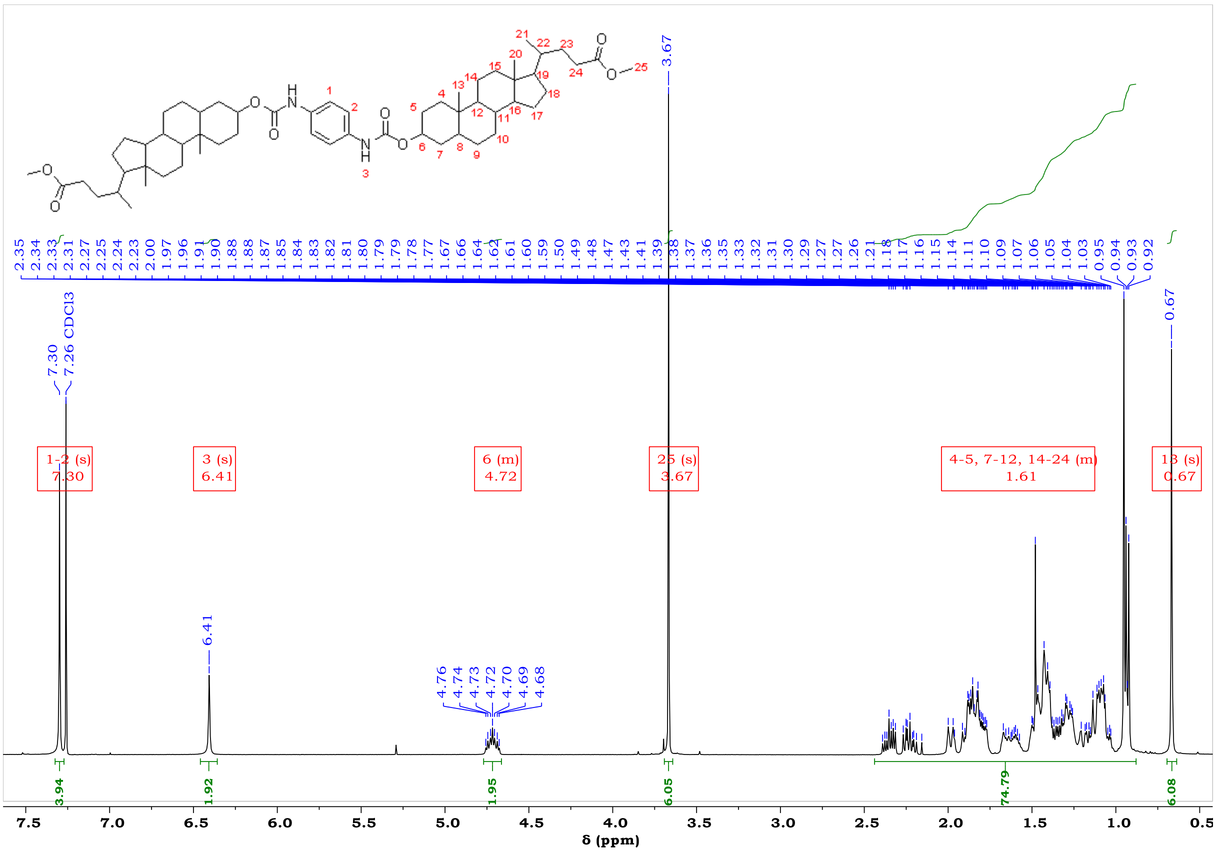

Figure S1. ^{1}H NMR spectrum of bischolesterol diester **2**.

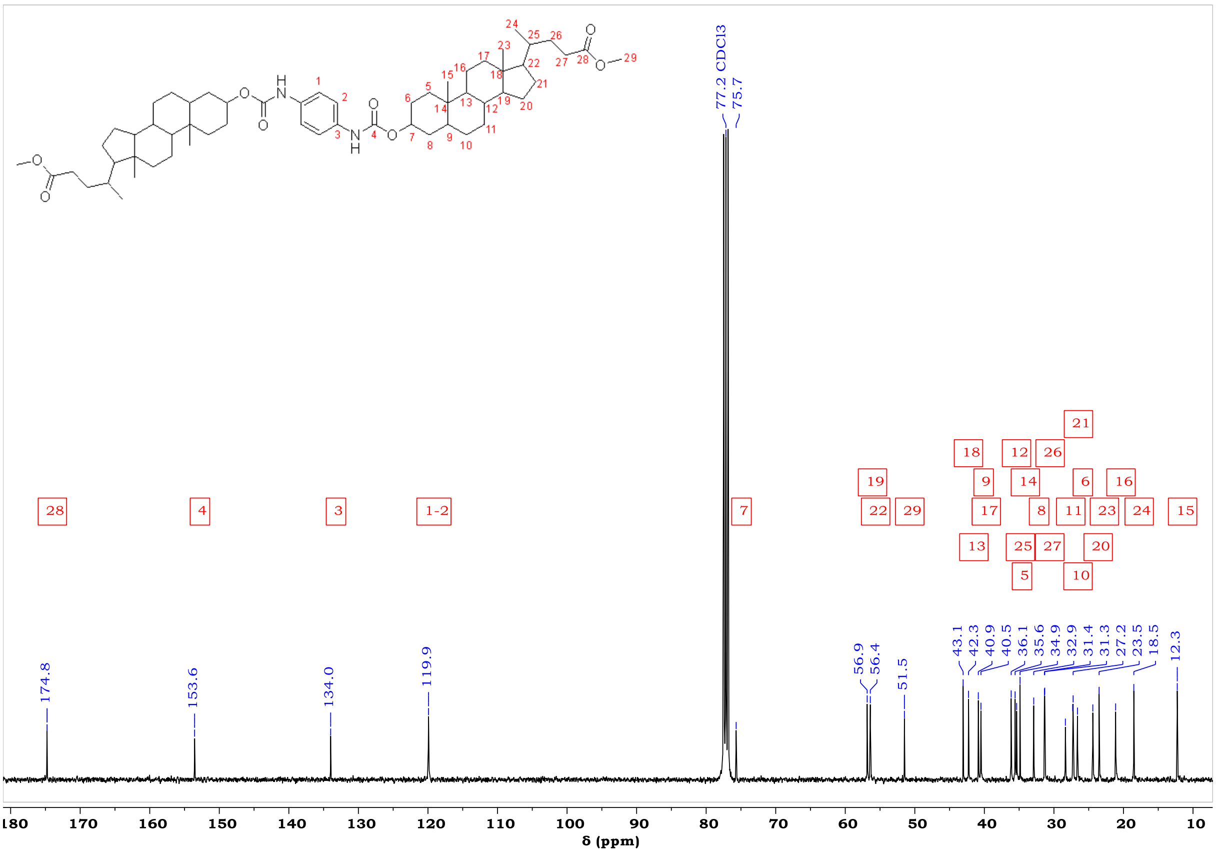

Figure S2. ^{13}C NMR spectrum of bischolesterol diester **2**.

Synthesis of bischolesterol dialcohol 3

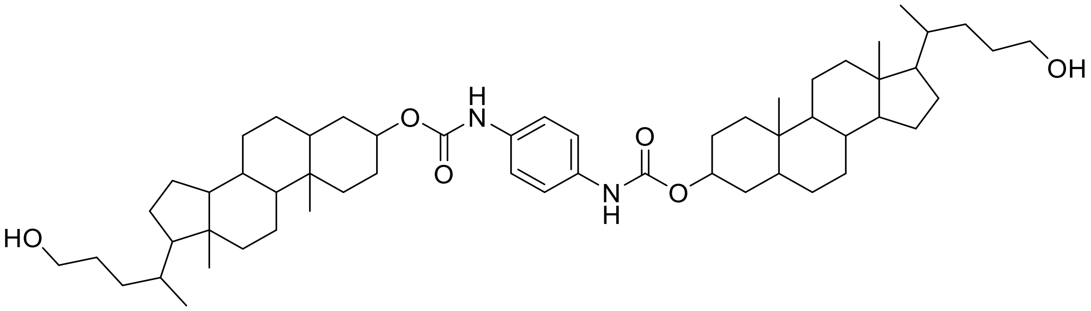

Adapted from Kobuke[3]

Bischolesterol diester **2** (558mg, 0.59mmol) was suspended in dry THF (25ml) under argon and heated to 50°C. $LiBH_4$ (50mg, 2.30mmol) was then added, and the reaction heated to 60°C and left to stir at this temperature for 4 hours. The reaction was cooled to RT and left stirring under argon overnight. 1N aqueous HCl was added dropwise until effervescence had ceased, and the reaction mixture was diluted with water, extracted with chloroform, and dried over $MgSO_4$. After evaporation of the solution, the residue was purified by column chromatography (4% methanol in chloroform) to yield the final product (450mg, 86%).

^{1}H NMR (400MHz, $CDCl_3$) δ 7.30 (s, 4H), 6.53 (s, 2H), 4.74-4.66 (m, 2H), 3.63-3.58 (m, 4H), 1.99-0.65 (m, 77H), 0.65 (s, 6H). ^{13}C NMR (100MHz, $CDCl_3$) δ 153.5 (C=O), 133.8 (Ar-C), 119.6 (Ar-CH), 75.5 (CH), 63.8 (CH_2), 56.7 (CH), 56.4 (CH), 42.9 (C), 42.1 (CH), 40.6 (CH), 40.3 (CH_2), 36.0 (CH), 35.7 (CH), 35.2 (CH_2), 34.7 (C), 32.7 (CH_2), 32.0 (CH_2), 29.5 (CH_2), 28.5 (CH_2), 27.2 (CH_2), 27.1 (CH_2), 26.5 (CH_2), 24.4 (CH_2), 23.5 (CH_3), 21.0 (CH_2), 18.8 (CH_3), 12.2 (CH_3). HRMS (TOF-ESI +ve) (*m/z*): $[M+Na]^+$ calcd for $C_{56}H_{88}N_2O_6Na$, 907.6540; found 907.6541. IR neat (cm^{-1}): 3424 (w, OH), 2938 (s, CH, aliphatic), 2865 (s, CH, aliphatic), 1720 (s, C=O, carbamate), 1624 (s, C=C, aromatic). Melting point: 203°C.

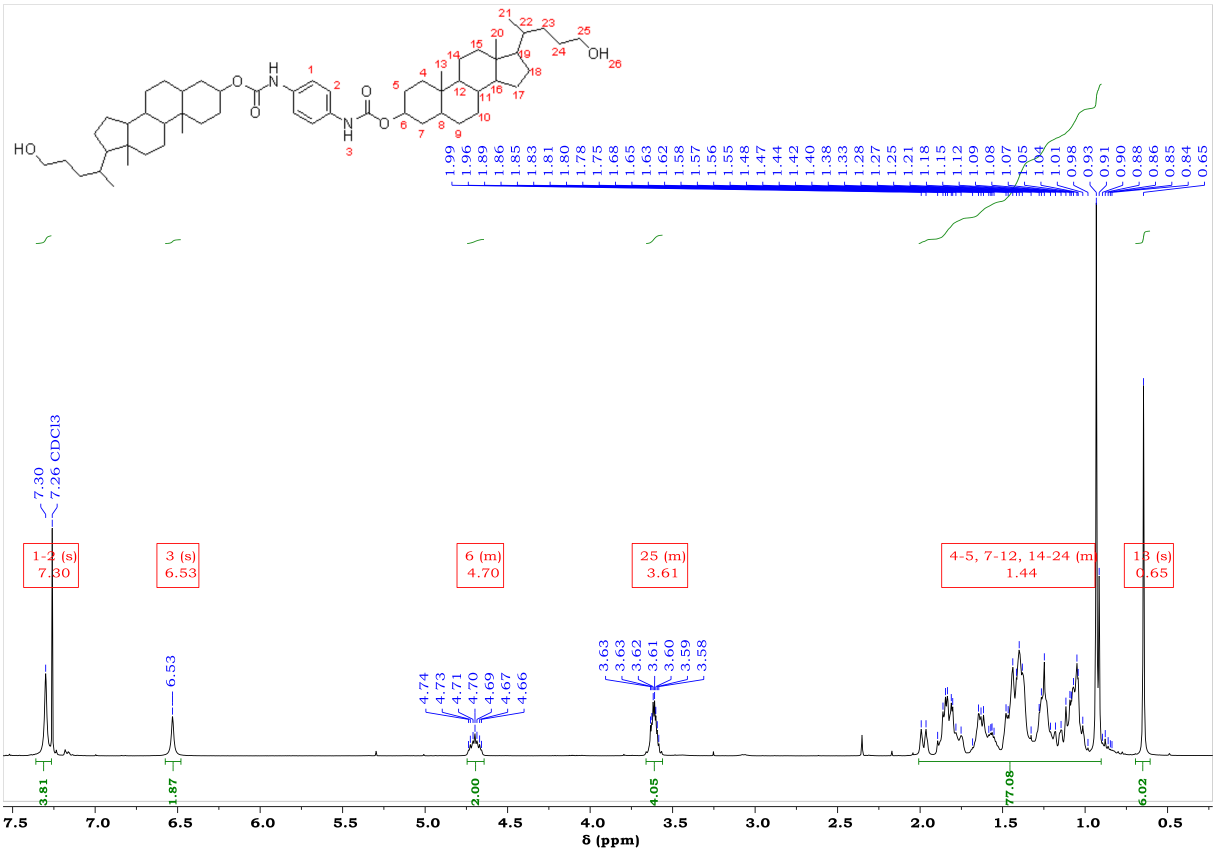

Figure S3. ^{1}H NMR spectrum of bischolesterol dialcohol **3**.

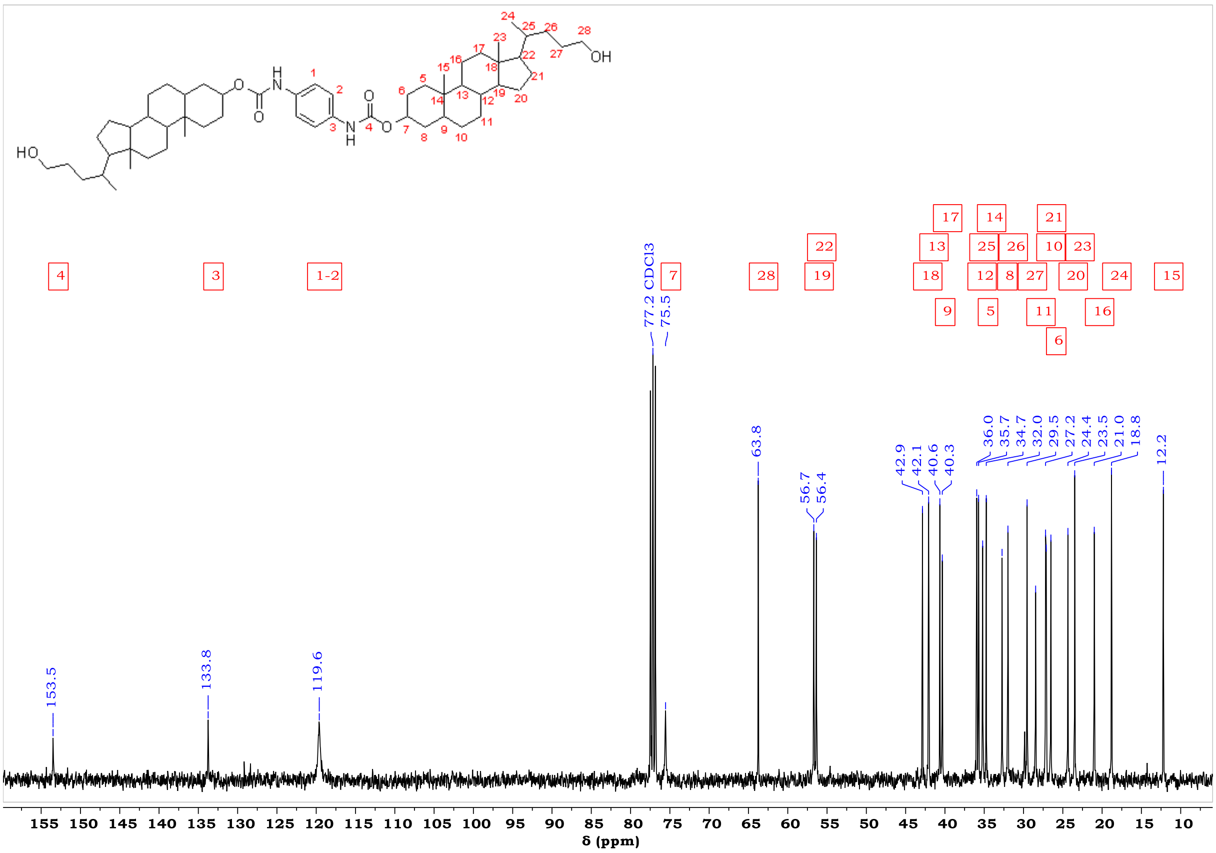

Figure S4. ^{13}C NMR spectrum of bischolesterol dialcohol **3**.

Synthesis of bischolesterol dibromide 4

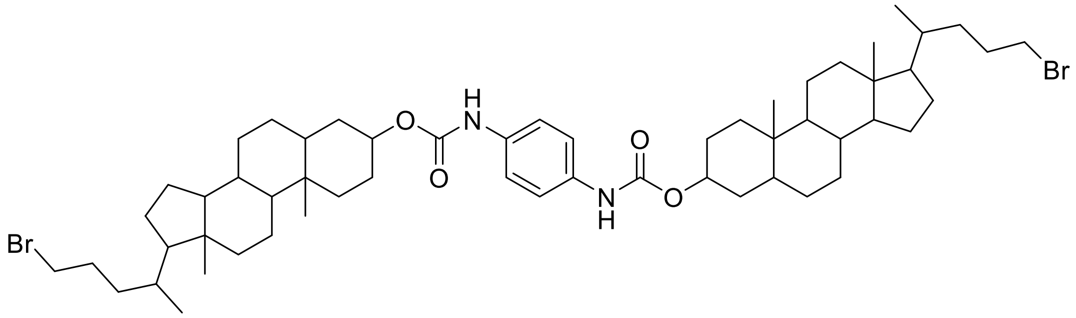

Under an inert atmosphere, pyridine (0.2ml, 2.58mmol) was added to a solution of triphenylphosphine (1.58g, 6.01mmol) and *N*-bromosuccinimide (1.15g, 6.44mmol) in dry DCM (20ml). A solution of bischolesterol dialcohol **3** (950mg, 1.07mmol) in dry DCM (50ml) was then added, and the resulting reaction was left stirring under argon at RT for 3 days. The solvent was removed, co-evaporating with toluene (3 x 100ml), to give a black/brown residue which was purified by column chromatography (1:1 toluene:ethyl acetate) to yield the final product as an off white solid (603mg, 56%).

^{1}H NMR (400MHz, $CDCl_3$) δ 7.30 (s, 4H), 6.44 (s, 2H), 4.73-4.67 (m, 2H), 3.44-3.33 (m, 4H), 1.99-0.88 (m, 70H), 0.65 (s, 6H). ^{13}C NMR (100MHz, $CDCl_3$) δ 153.5 (C=O), 133.8 (Ar-C), 119.6 (Ar-CH), 75.5 (CH), 56.7 (CH), 56.2 (CH), 42.9 (C), 42.1 (CH), 40.6 (CH), 40.3 (CH_2), 36.0 (CH), 35.4 (CH), 35.2 (CH_2), 34.7 (CH_2), 34.7 (C), 32.7 (CH_2), 29.8 (CH_2), 28.4 (CH_2), 27.2 (CH_2), 27.1 (CH_2), 26.5 (CH_2), 24.3 (CH_2), 23.5 (CH_3), 21.0 (CH_2), 18.8 (CH_3), 12.2 (CH_3). HRMS (TOF-ESI +ve) (*m/z*): $[M+Na]^+$ calcd for $C_{56}H_{88}N_2O_4Na^{79}Br^{81}Br$, 1033.4832; found 1033.4838. IR neat (cm^{-1}): 3321 (w, NH, carbamate), 2936 (s, CH, aliphatic), 2865 (s, CH, aliphatic), 1726 (m, C=O, carbamate), 1704 (m, C=O, carbamate), 1524 (m, C=C, aromatic). Melting point: 133-134°C.

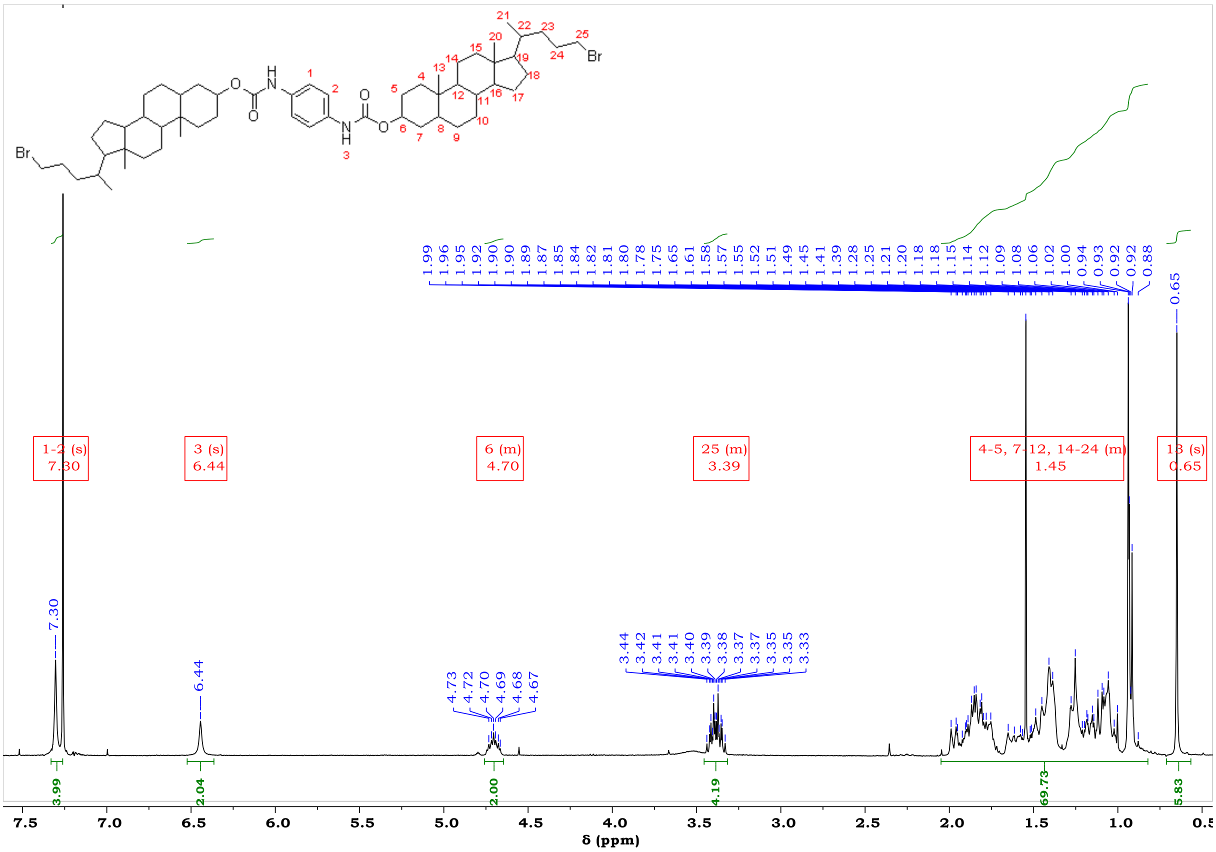

Figure S5. ^{1}H NMR spectrum of bischolesterol dibromide **4**.

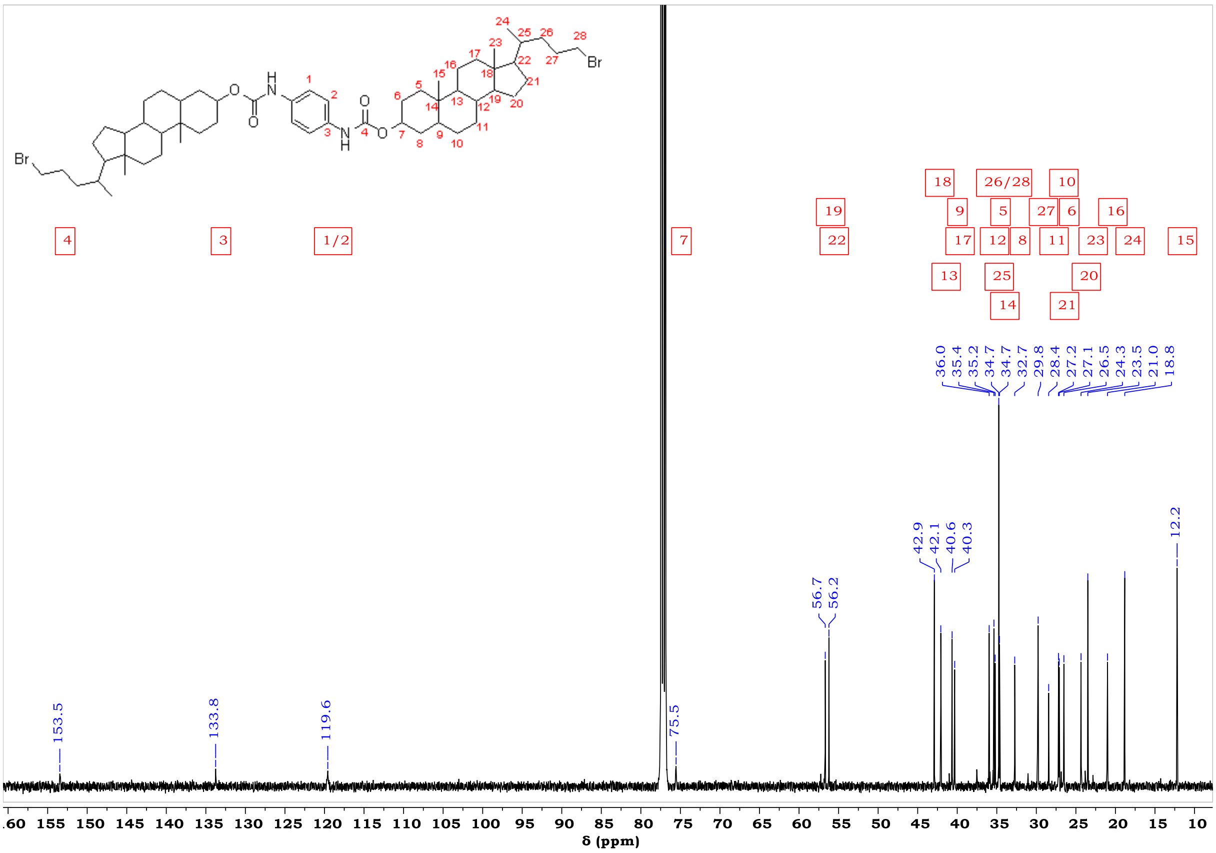

Figure S6. ^{13}C NMR spectrum of bischolesterol dibromide **4**.

Synthesis of bischolesterol diazide 5

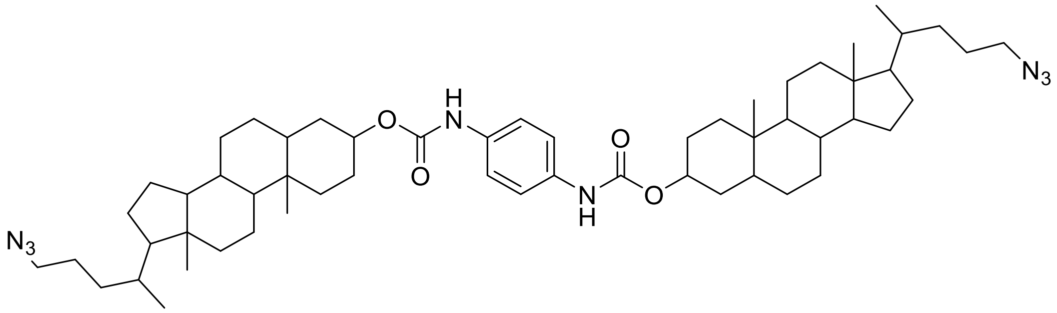

Adapted from Sharma and Gilmer[4]

To a stirred solution of bischolesterol dibromide **4** (560mg, 0.55mmol) in anhydrous DMF (16ml) was added, sodium azide (216mg, 3.3mmol) and sodium iodide (17mg, 0.11mmol). The reaction was heated to 60°C and left to stir at this temperature under argon for 2 days. Water (50ml) was added to precipitate the final product, which was isolated by filtration and washed with water to give an off white solid (290mg, 59%).

^{1}H NMR (400MHz, $CDCl_3$) δ 7.30 (s, 4H), 6.48 (s, 2H), 4.74-4.66 (m, 2H), 3.29-3.17 (m, 4H), 1.99-0.92 (m, 80H), 0.65 (s, 6H). ^{13}C NMR (100MHz, $CDCl_3$) δ 153.5 (C=O), 133.8 (Ar-C), 119.6 (Ar-CH), 75.6 (CH), 56.7 (CH), 56.2 (CH), 52.1 (CH_2), 42.9 (C), 42.1 (CH), 40.6 (CH), 40.3 (CH_2), 36.0 (CH), 35.6 (CH), 35.2 (CH_2), 34.7 (C), 33.1 (CH_2), 32.7 (CH_2), 28.4 (CH_2), 27.2 (CH_2), 27.1 (CH_2), 26.5 (CH_2), 25.7 (CH_2), 24.3 (CH_2), 23.5 (CH_3), 21.0 (CH_2), 18.8 (CH_3), 12.2 (CH_3). HRMS (TOF-ESI +ve) (*m/z*): $[M+Na]^+$ calcd for $C_{56}H_{86}N_8O_4Na$, 957.6670; found 957.6671. IR neat (cm^{-1}): 3322 (w, NH, carbamate), 2930 (s, CH, aliphatic), 2865 (s, CH, aliphatic), 2092 (m, N=N=N, azide), 1710 (m, C=O, carbamate), 1523 (s, C=C, aromatic). Melting point: 196-198°C.

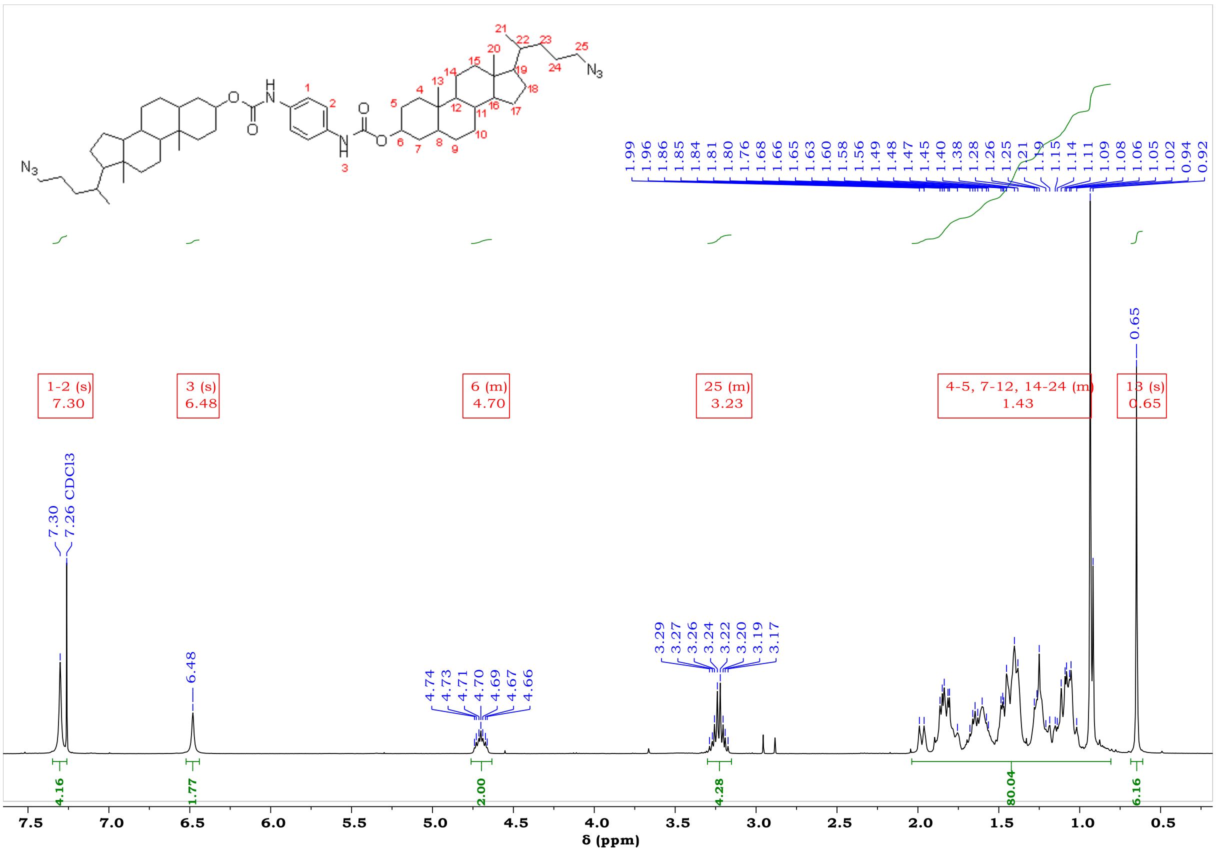

Figure S7. ^{1}H NMR spectrum of bischolesterol diazide **5**.

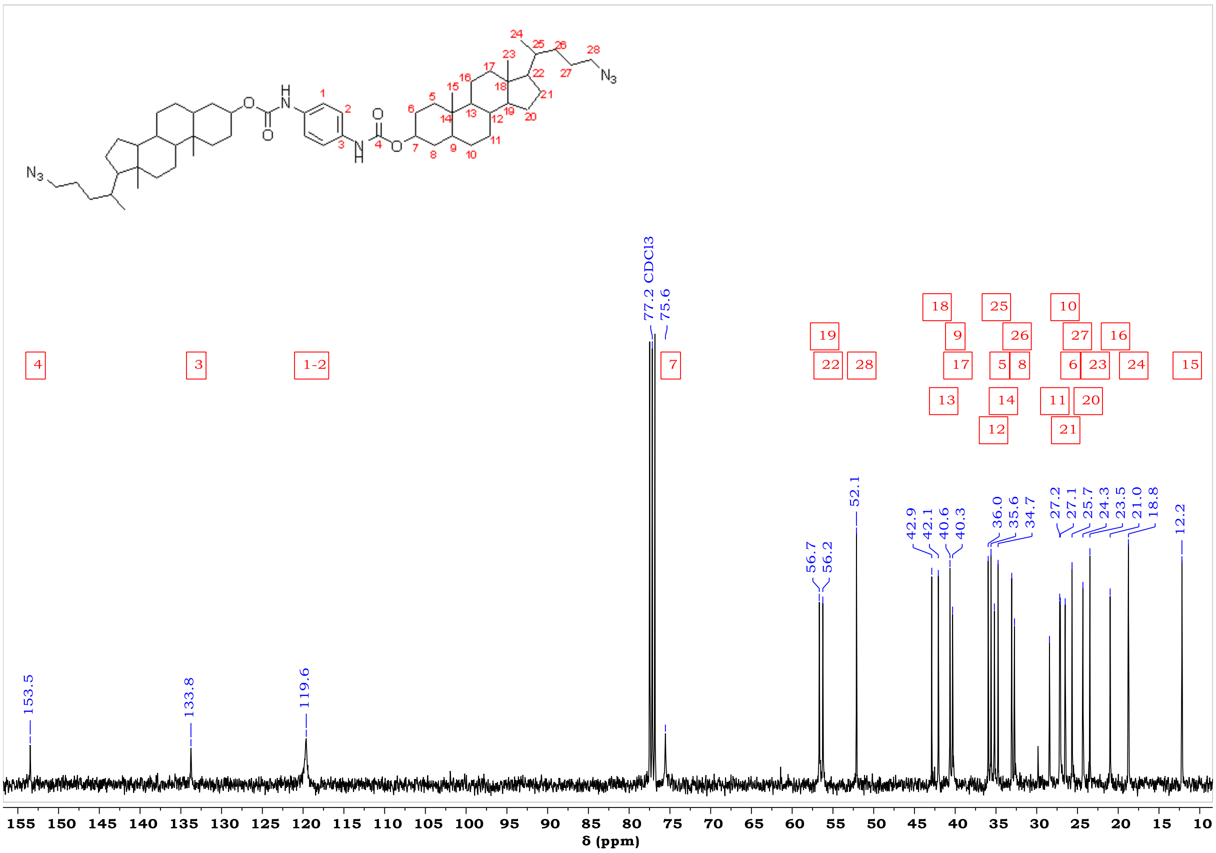

Figure S8. ^{13}C NMR spectrum of bischolesterol diazide **5**.

Crystal structure determination of bischolesterol diester 2, bischolesterol dibromide 4, and bischolesterol diazide 5

The datasets were measured on an Agilent SuperNova diffractometer using an Atlas detector. The data collections were driven and processed, and absorption corrections was applied using CrysAlisPro. The structures of bischolesterol diester **2** and bischolesterol dibromide **4** were solved using ShelXS[5] while the structure of bischolesterol diazide **5** was solved using ShelXT.[6] All three structures were refined by a full-matrix least-squares procedure on F^2 in ShelXL.[6] All non-hydrogen atoms were refined with anisotropic displacement parameters. In bischolesterol diester **2**, the hydrogen atoms bonded to N(1) and N(2) were located in the electron density and their positions refined freely, with their U(iso) values based on the U(eq) values of the parent atoms. All remaining hydrogen atoms in bischolesterol diester **2** and all hydrogen atoms in bischolesterol dibromide **4** and bischolesterol diazide **5** were fixed as riding models and the isotropic thermal parameters (U_{iso} based on the U_{eq} of the parent atom. Figures and reports were produced using OLEX2.[7] CCDC 2050123-2050125 contain the supplementary crystallographic data for bischolesterol diester **2**, bischolesterol dibromide **4** and bischolesterol diazide **5**, respectively. These data can be obtained free of charge from The Cambridge Crystallographic Data Centre *via* www.ccdc.cam.ac.uk/data_request/cif.

All three structures occupy chiral space groups. For bischolesterol diester **2** and bischolesterol diazide **5**, it was not possible to determine the absolute structure from the diffraction data. However, for bischolesterol dibromide **4** the Flack parameter was determined as -0.005(10) which means that the absolute structure can confidently be assigned from the diffraction data.

The structure of bischolesterol diester **2** contains a molecule of toluene, (C(101)-C(107) and C(11’)-C(17’)) which is disordered over two positions at a refined percentage occupancy ratio of 61.7(11):38.3(11) respectively.

The structure of bischolesterol dibromide **4** contains three crystallographically-independent molecules and six molecules of DMF. One of the bromine atoms belonging to molecule 2 (C(101)-C(156), N(101)-N(102), O(101)-O(104), Br(11)-

Br(12)) is disordered over two positions labelled Br(12) / Br(2') at a refined occupancy ratio 89.7(5):10.3(5) respectively. In molecule 3 (C(201)-C(256), N(201)-N(202), O(201)-O(204), Br(21)-Br(22)) the C(255), C(256), Br(22) / C(55'), C(56'), Br(3)' group is disordered over two positions at a refined occupancy ratio of 67.7(6): 32.3(6). One of the six molecules of DMF that are present, (C(801)-C(803), N(801), O(801) / C(81')-C(83'), N(81'), O(81')) is disordered over two positions with a refined percentage occupancy ratio of 63.7(17):36.3(17).

The structure of bischolesterol diazide **5** contains two molecules of DMF, (C(101)-C(103), N(101), O(101) / C(11')-C(13'), N(11'), O(11') and C(201)-C(203), N(201), O(201) / C(21')-C(23'), N(21'), O(21')) which are both disordered over two positions with a refined percentage occupancy ratios of 71.6(6):28.4(6) and 54.1(5): 45.9(5) respectively.

Crystal data for bischolesterol diester 2

$C_{65}H_{96}N_2O_8$ (M =1033.43g/mol): triclinic, space group P1 (no. 1), a = 7.8291(3)Å, b = 10.2983(4)Å, c = 18.1014(7)Å, α = 97.459(3)°, β = 92.901(3)°, γ = 92.677(3)°, V = 1443.16(9)Å^3, Z = 1, T = 99.95(18)K, μ(CuKα) = 0.602mm^{-1}, $Dcalc$ = 1.189g/cm^3, 23539 reflections measured (8.672° ≤ 2Θ ≤ 144.194°), 10702 unique (R_{int} = 0.0332, R_{sigma} = 0.0384) which were used in all calculations. The final R_1 was 0.0562 (I > 2σ(I)) and wR_2 was 0.1690 (all data).

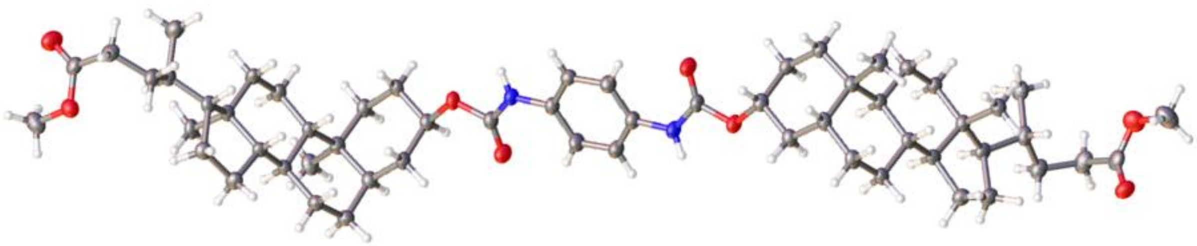

Figure S9. Crystal structure of bischolesterol diester **2** with ellipsoids drawn at the 50% probability level. The structure contains a molecule of toluene which has been omitted for clarity.

Crystal data for bischolesterol dibromide 4

$C_{62}H_{100}Br_2N_4O_6$ (M =1157.27g/mol): monoclinic, space group P2$_1$ (no. 4), a = 8.68918(6)Å, b = 59.9830(5)Å, c = 17.67491(16)Å, β = 97.1524(7)°, V = 9140.54(13)Å^3, Z = 6, T = 100.01(10)K, μ(CuKα) = 2.084mm^{-1}, $Dcalc$ = 1.261g/cm^3,

82168 reflections measured (5.25° ≤ 2Θ ≤ 136.502°), 32056 unique (R_{int} = 0.0734, R_{sigma} = 0.0718) which were used in all calculations. The final R_1 was 0.0764 (I > 2σ(I)) and wR_2 was 0.2006 (all data).

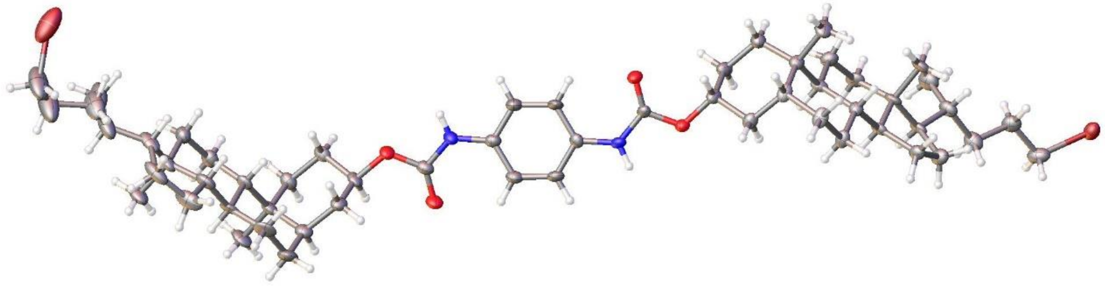

Figure S10. Crystal structure of molecule 1 of bischolesterol dibromide **4** with ellipsoids drawn at the 50% probability level. The structure contains three crystallographically-independent molecules and six molecules of DMF, of which only molecule 1 is shown.

Crystal data for bischolesterol diazide 5

$C_{62}H_{100}N_{10}O_6$ (*M* =1081.51g/mol): orthorhombic, space group $P2_12_12_1$ (no. 19), *a* = 8.85680(10)Å, *b* = 20.1304(3)Å, *c* = 34.4364(5)Å, *V* = 6139.70(15)Å^3, *Z* = 4, *T* = 100.00(10)K, μ(CuKα) = 0.599mm^{-1}, *Dcalc* = 1.170g/cm^3, 40102 reflections measured (8.786° ≤ 2Θ ≤ 147.748°), 12232 unique (R_{int} = 0.0329, R_{sigma} = 0.0252) which were used in all calculations. The final R_1 was 0.0942 (I > 2σ(I)) and wR_2 was 0.2751 (all data).

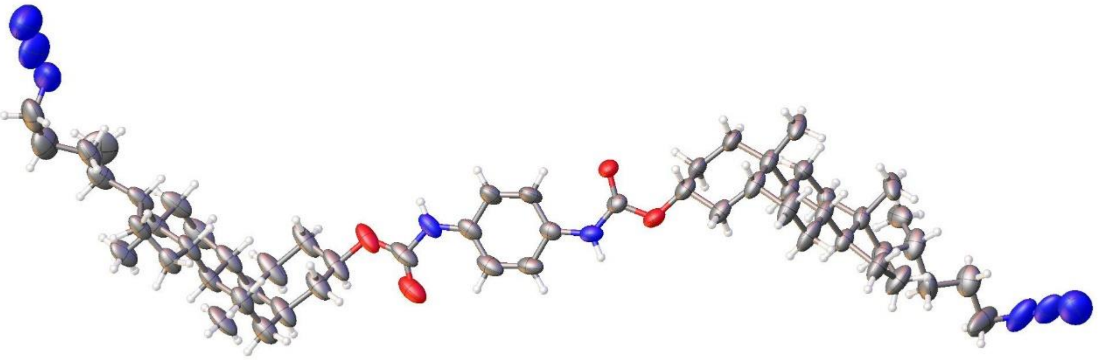

Figure S11. Crystal structure of bischolesterol diazide **5** with ellipsoids drawn at the 50% probability level. The structure contains two molecules of DMF which have been omitted for clarity.

General procedure for the synthesis of bischolesterol dye conjugates

Equimolar equivalents of bischolesterol diazide and the corresponding cyanine dye were dissolved in dry THF in an inert atmosphere. DIPEA and copper iodide were then added, and the reaction was left stirring at room temperature for 12 hours. The reaction was quenched with water, and the product extracted with DCM. Organics were combined and dried over magnesium sulphate which was subsequently removed by filtration along with undissolved copper iodide. The solvent was removed to leave crude product, which was then purified by either flash chromatography, HPLC, or both techniques.

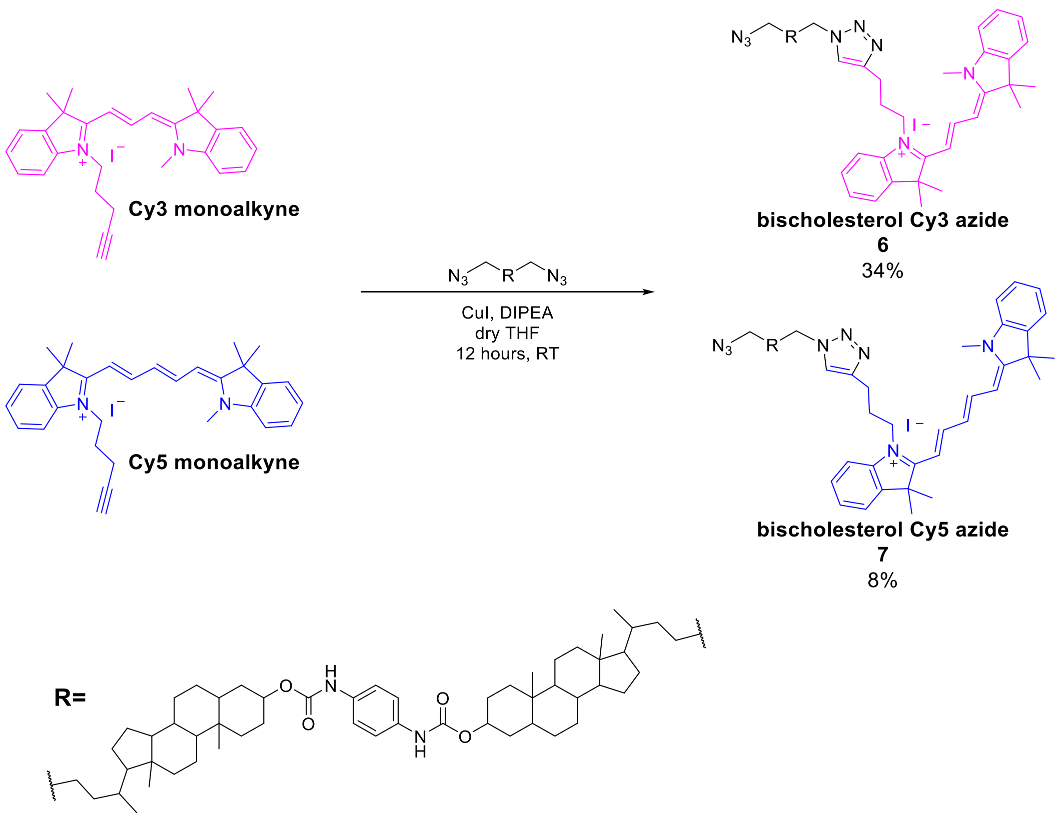

Scheme S2. Synthesis of the bischolesterol dye conjugates.

HPLC purification of the bischolesterol dye conjugates

HPLC purification of the conjugates was performed on a Kinetex C18 prep column from Phenomenex, following the method described in the table below:

Table S1. Solvent gradients used in the HPLC purification of the bischolesterol dye conjugates.

Time/mins	Water + 0.1% TFA/%	Methanol + 0.1% TFA/%
0	10	90
10	10	90
20	0	100
30	0	100
30.1	10	90
35	10	90

HPLC purification of Cy3 containing products were monitored at 260nm and 550nm whereas Cy5 containing products were monitored at 260nm and 645nm. Desired products eluted at ~25 minutes.

Synthesis of bischolesterol Cy3 azide 6

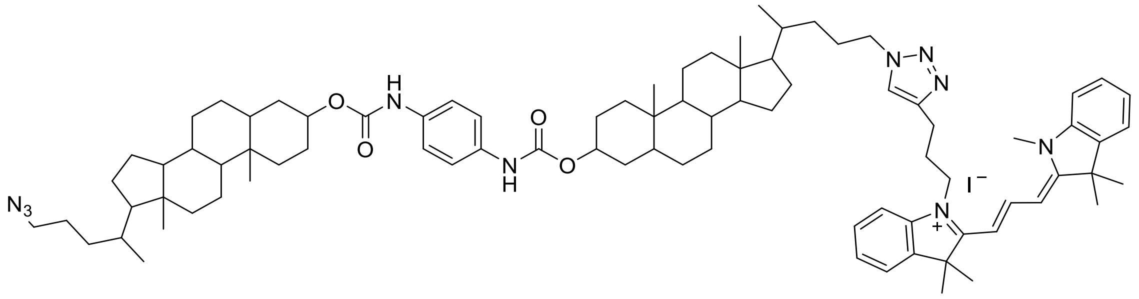

Bischolesterol Cy3 azide was synthesised in accordance to the general procedure, using bischolesterol diazide **5** (39mg, 0.04mmol), Cy3 monoalkyne (22.6mg, 0.04mmol), DIPEA (7µl, 0.004mmol), and copper iodide (4mg, 0.02mmol) in dry THF (5ml). The crude was purified by the HPLC purification procedure described above to yield the final product as a pink solid (21mg, 34%).

^{1}H NMR (400MHz, $CDCl_3$) δ 8.38 (t, J= 13.2Hz, 1H), 7.89 (s, 1H), 7.43-7.24 (m, 12H), 7.12 (d, J= 7.9Hz, 1H), 6.58-6.51 (m, 4H), 4.73-4.66 (m, 2H), 4.37-4.26 (m, 2H), 4.17 (s, 2H), 3.66 (s, 3H), 3.29-3.17 (m, 2H), 3.04 (s, 2H), 2.26 (s, 2H), 1.99-0.83 (m, 84H), 0.65 (s, 3H), 0.61 (s, 3H). ^{13}C NMR (100MHz, $CDCl_3$) δ 174.5 (C), 174.2 (C), 153.5 (C=O), 150.7 (H*C*=C), 142.8 (Ar-C), 142.0 (Ar-C), 140.5 (Ar-C), 140.5 (Ar-C), 133.8 (Ar-C), 129.4

(Ar-CH), 129.1 (Ar-CH), 125.8 (Ar-CH), 125.6 (Ar-CH), 122.2 (H*C*=C), 122.2 (Ar-CH), 119.6 (Ar-CH), 111.7 (Ar-CH), 110.9 (Ar-CH), 104.0 (H*C*=C), 103.9 (H*C*=C), 75.6 (CH), 56.7 (CH), 56.6 (CH), 56.2 (CH), 55.9 (CH), 52.1 (CH_2), 51.7 (CH_2), 49.3 (C), 49.1 (C), 44.1 (CH_2), 42.9 (C), 42.1 (CH), 40.6 (CH), 40.3 (CH_2), 36.0 (CH), 35.6 (CH), 35.4 (CH), 35.2 (CH_2), 34.7 (C), 33.1 (CH_2), 32.7 (CH_2), 31.4 (NCH_3), 29.8 (CH_2), 28.4 (CH_2), 28.2 (CH_3), 27.2 (CH_2), 27.1 (CH_2), 26.8 (CH_2), 26.5 (CH_2), 25.7 (CH_2), 24.3 (CH_2), 23.5 (CH_3), 22.8 (CH_2), 21.0 (CH_2), 18.8 (CH_3), 18.6 (CH_3), 12.2 (CH_3). HRMS (TOF-ESI +ve) (*m/z*): $[M-I]^+$ calcd for $C_{85}H_{119}N_{10}O_4$, 1344.9416; found 1344.9415.

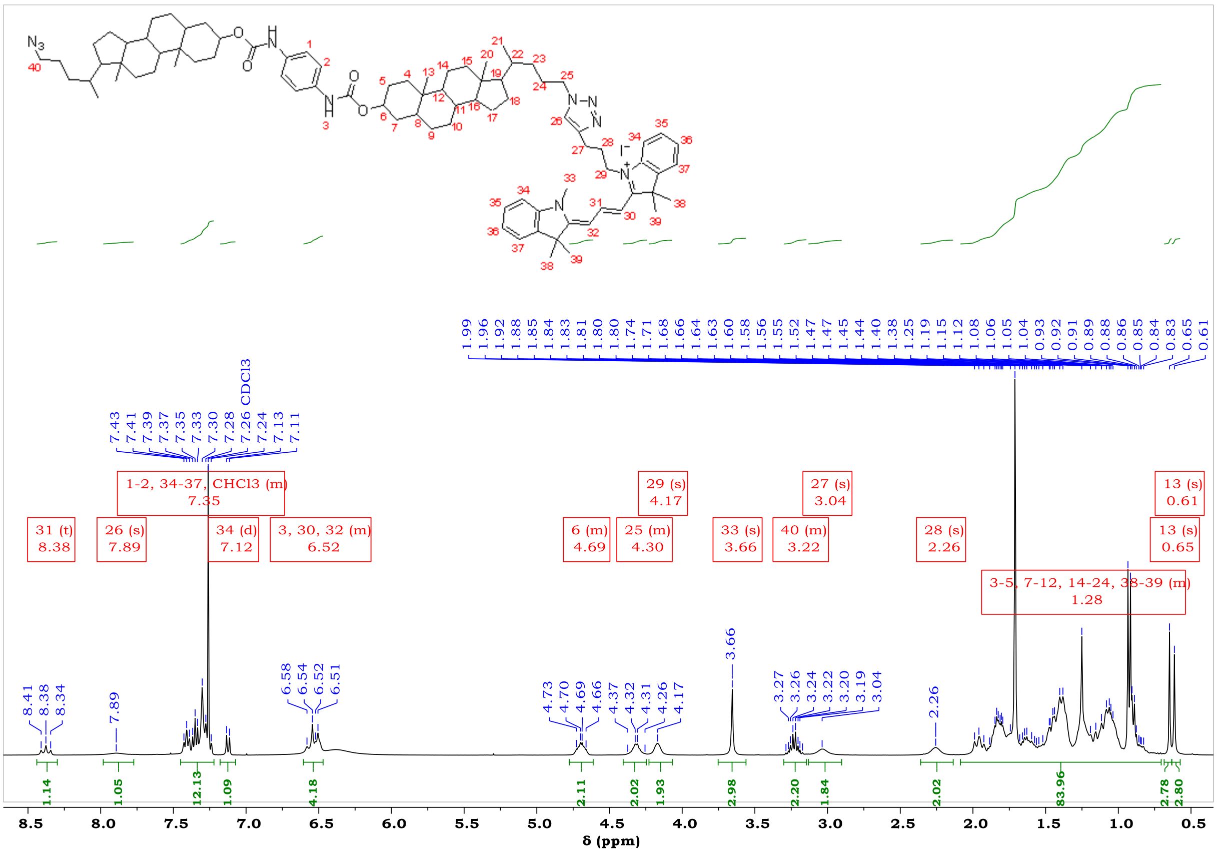

Figure S12. ^{1}H NMR spectrum of bischolesterol Cy3 azide **6**.

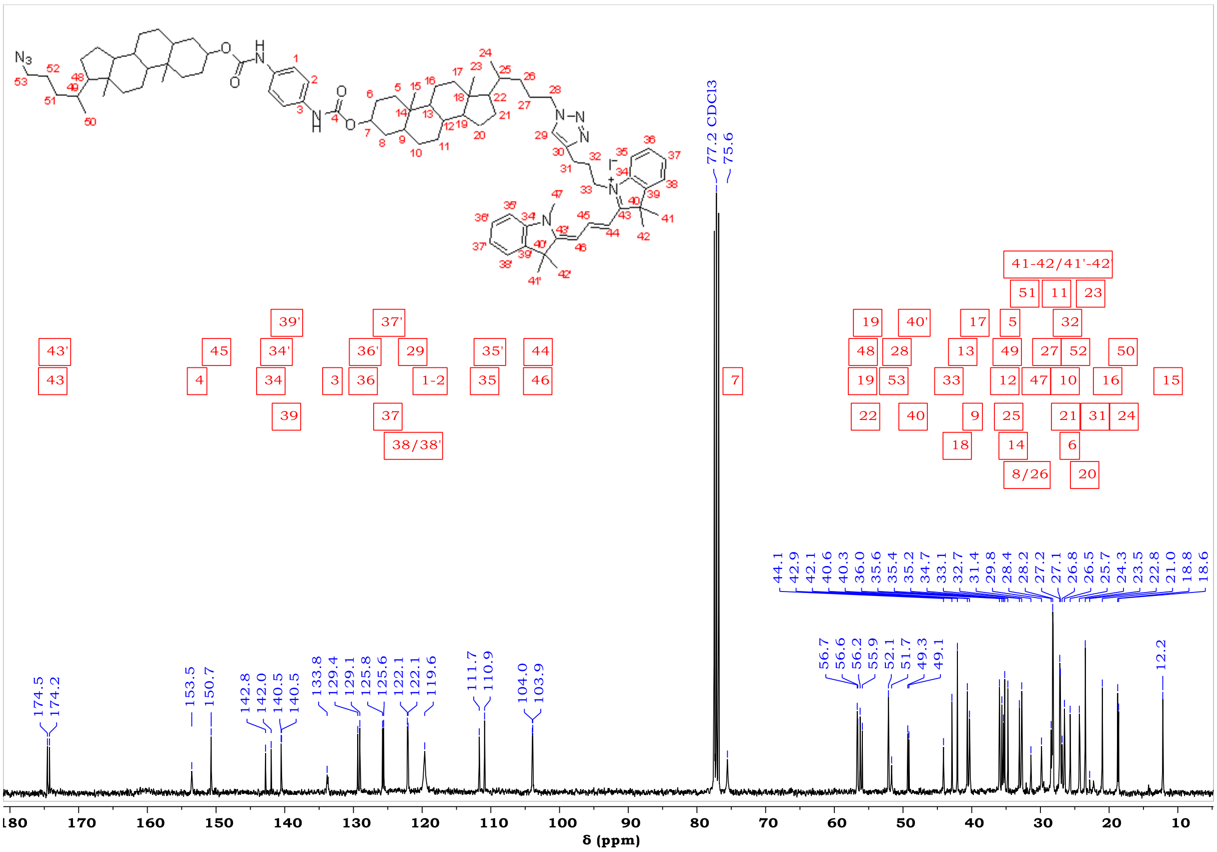

Figure S13. ^{13}C NMR spectrum of bischolesterol Cy3 azide **6**.

<u>Synthesis of bischolesterol Cy5 azide 7</u>

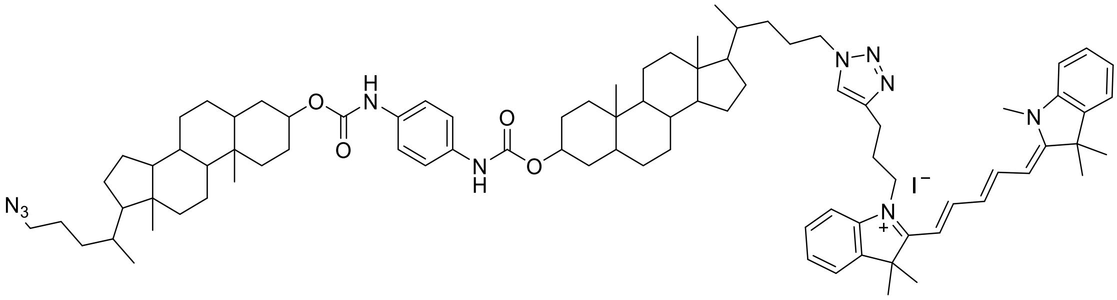

Bischolesterol Cy5 azide **7** was synthesised in accordance to the general procedure, using bischolesterol diazide **5** (50mg, 0.05mmol), Cy5 monoalkyne (30mg, 0.05mmol), DIPEA (9µl, 0.005mmol), and copper iodide (5mg, 0.025mmol) in dry THF (5ml). The crude was purified by flash chromatography with an eluent system of 95:5 DCM:MeOH and further purified by the HPLC purification procedure described above to yield the final product as a blue solid (13mg, 8%).

^{1}H NMR (400MHz, $CDCl_3$) δ 7.84-7.77 (m, 3H), 7.39-7.21 (m, 15H), 7.07 (d, J= 7.9Hz, 1H), 6.67 (s, 1H), 6.54 (s, 1H), 6.48 (s, 1H), 6.35 (d, J= 13.3Hz, 1H), 6.19 (d, J= 13.0Hz,

1H), 4.72-4.67 (m, 2H), 4.32 (s, 2H), 4.14 (s, 2H), 3.58 (s, 3H), 3.28-3.18 (m, 2H), 3.00 (s, 2H), 2.24 (s, 2H), 1.99-0.83 (m, 86H), 0.65 (s, 3H), 0.62 (s, 3H). ^{13}C NMR (100MHz, $CDCl_3$) δ 173.3 (C), 172.9 (C), 153.6 (H*C*=C), 153.6 (C=O), 152.9 (H*C*=C), 145.1 (C), 142.9 (Ar-C), 142.1 (Ar-C), 141.1 (Ar-C), 140.8 (Ar-C), 133.9 (Ar-C), 129.1 (Ar-CH), 128.8 (Ar-CH), 126.5 (H*C*=C), 125.6 (Ar-CH), 125.2 (Ar-CH), 122.2 (H*C*=C/Ar-CH), 119.7 (Ar-CH), 111.3 (Ar-CH), 110.4 (Ar-CH), 104.5 (H*C*=C), 103.7 (H*C*=C), 75.6 (CH), 56.7 (CH), 56.6 (CH), 56.3 (CH), 55.9 (CH), 52.1 (CH_2), 51.4 (CH_2), 49.5 (C), 49.2 (C), 44.0 (CH_2), 42.9 (C), 42.1 (CH), 40.7 (CH), 40.3 (CH_2), 36.0 (CH), 35.6 (CH), 35.4 (CH), 35.2 (CH_2), 34.8 (C), 33.1 (CH_2), 32.7 (CH_2), 31.3 (NCH_3), 29.8 (CH_2), 28.4 (CH_2), 28.1 (CH_3), 27.2 (CH_2), 27.1 (CH_2), 26.9 (CH_2), 26.5 (CH_2), 25.7 (CH_2), 24.3 (CH_2), 23.5 (CH_3), 22.4 (CH_2), 21.0 (CH_2), 18.8 (CH_3), 18.7 (CH_3), 12.2 (CH_3). HRMS (TOF-ESI +ve) (*m/z*): $[M-I]^+$ calcd for $C_{87}H_{121}N_{10}O_4$, 1370.9572; found 1370.9568.

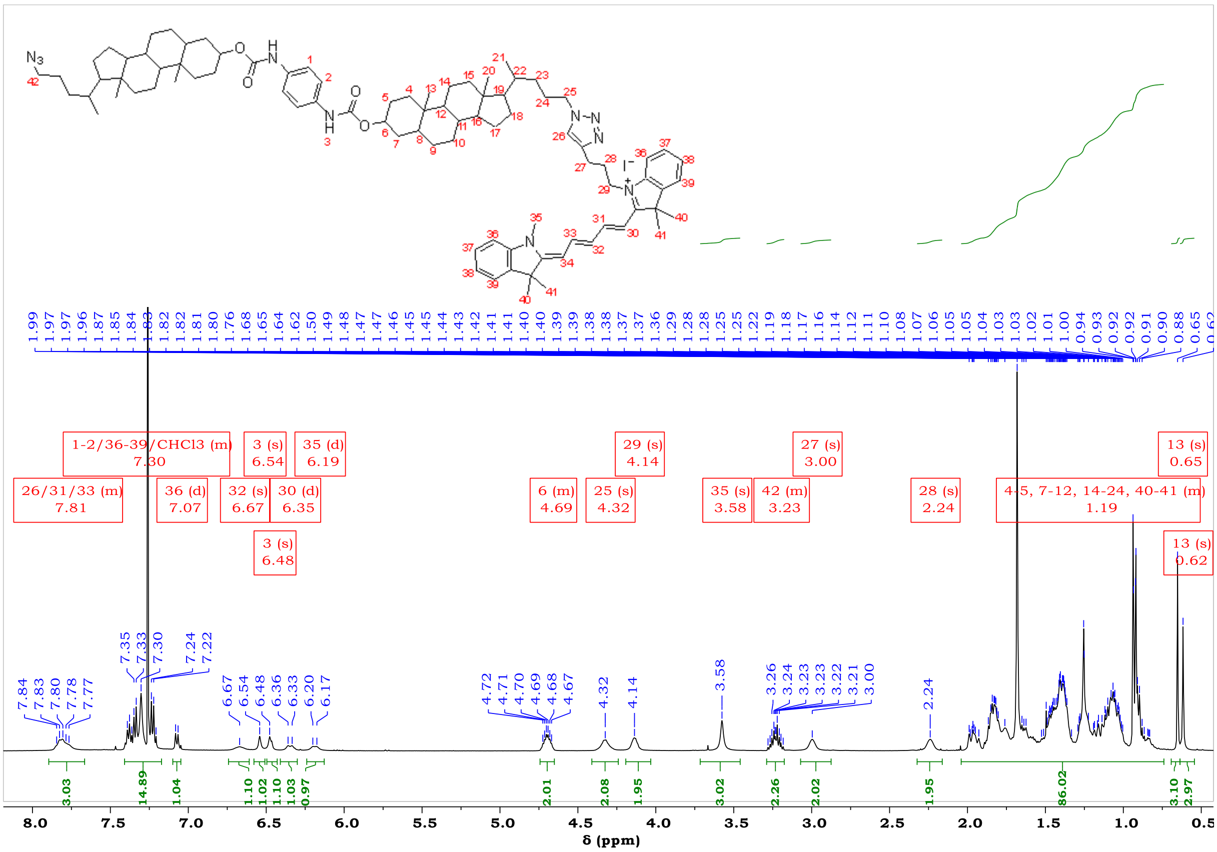

Figure S14. ^{1}H NMR spectrum of bischolesterol Cy5 azide **7**.

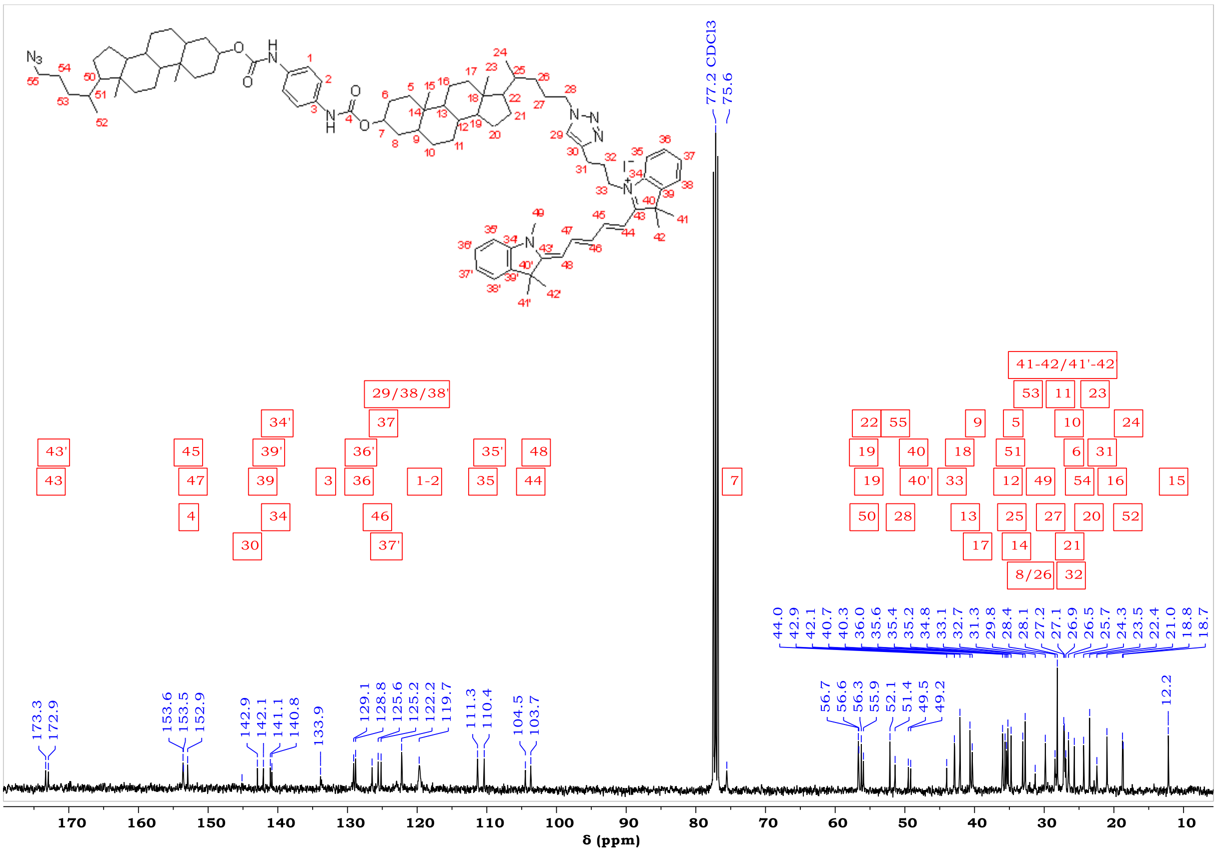

Figure S15. ^{13}C NMR spectrum of bischolesterol Cy5 azide **7**.

Synthesis and purification of oligonucleotides

Oligonucleotides were synthesised using solid phase synthesis on an Applied Biosystems ABI 394 DNA/RNA synthesiser using commercially supplied DNA synthesis grade solvents and reagents.

Standard synthesis of complementary, half-complementary, scrambled, and alkyne modified strands

Standard phosphoramidites of Bz-dA, iBu-dG, Ac-dC, and dT from Link Technologies, and 5'-hexynyl phosphoramidite from Glen Research were used for synthesis. The phosphoramidites were dissolved in anhydrous acetonitrile to 0.1M prior to synthesis. Strands were synthesised at a 1µmol scale on SynBase™ CPG 1000/110 solid supports from Link Technologies. The resins have average pore sizes of 1000Å, nominal particle sizes of 110µm, and nucleoside loadings of 25-40µmol/g. Phosphoramidites were activated with 5-ethylthio-1H-tetrazole (0.5M) in acetonitrile prior to coupling. Coupling times of 25 seconds and 10 minutes were used for the nucleosides and hexynyl group respectively. Acetic anhydride and methylimidazole were added to cap unreacted material, and then iodine (0.1M) in THF/pyridine/water (78:20:2) was added to oxidise the phosphotriester formed. Upon sequence completion, oligonucleotides were treated with aqueous ammonia (30%) for 1 hour to cleave the strands from the resin. Protecting groups on the strands were removed by heating in aqueous ammonia (30%) at 60°C for 6 hours. The solvent was then removed on a Thermo Scientific speed vac in preparation for purification.

Ultramild synthesis of Cy3 and Cy5 modified control strands

Standard phosphoramidites of Pac-dA, iPr-Pac-dG, Ac-dC, dT, Cyanine-3-CE phosphoramidite, and Cyanine-5-CE phosphoramidite from Link Technologies were used for ultramild synthesis. The nucleoside phosphoramidites were dissolved in anhydrous acetonitrile to 0.1M prior to synthesis whereas Cyanine-3-CE phosphoramidite and Cyanine-5-CE phosphoramidite were dissolved to the same concentration in anhydrous DCM. Strands were synthesised at a 1µmol scale on SynBase™ CPG 1000/110 solid supports from Link Technologies. Phosphoramidites were activated with 5-ethylthio-1H-tetrazole (0.25M) in acetonitrile prior to coupling. Coupling times of 25 seconds was used for the nucleoside phosphoramidites, and 10 minutes for Cyanine-3-CE phosphoramidite

and Cyanine-5-CE phosphoramidite. Then, phenoxyacetic anhydride and methylimidazole were added to cap unreacted material, and iodine (0.02M) in THF/pyridine/water (7:2:1) was added to oxidise the phosphotriester formed. Upon sequence completion, the resins were placed in 1ml solutions of aqueous ammonia (30%) and shaken for 3 hours to cleave strands from the resin and remove protecting groups. The solutions were then desalted with a NAP-10 column from GE Healthcare, concentrated to 1ml on a Thermo Scientific speed vac, and stored in the freezer for purification.

Purification of complementary, half-complementary, scrambled, and alkyne modified strands

Semi preparative HPLC purification was performed using a Phenomenex Clarity 5μm Oligo-RP LC 250x10mm column. 1ml of sample was injected with a run time of 45 minutes for each sample, at a flow rate of 3ml/min. The column was heated to 60°C prior to sample injection. The UV/vis absorbance of each run was monitored at 260nm. The solvent gradients used are listed in the table below:

Table S2. Solvent gradients used in the HPLC purification of oligonucleotides.

Time/mins	0.1M TEAA in HPLC water/%	acetonitrile/%
0	95	5
30	82	18
30.1	0	100
40	0	100
40.1	95	5
45	95	5

Collected fractions were evaporated to dryness, diluted to 1ml in Milli-Q water, and desalted using a NAP-10 column (GE Healthcare), whilst eluting to 1.5ml. Purity of oligonucleotides was determined by analytical HPLC using a Phenomenex Clarity 5μm Oligo RP LC 250x4.6mm column. The column was heated to 60°C prior to sample injection. 20μl of sample was injected with a run time of 45 minutes for each sample, at a flow rate of 1ml/min. Solvent gradients used were identical to semi preparative HPLC (Table S2). The UV/vis absorbance of each run was monitored at 260nm.

Purification of Cy3 and Cy5 modified control strands

Cy3/Cy5 modified strands were purified and analysed with the same columns and systems as previous strands, with the exception of a different solvent gradient being applied to account for the enhanced lipophilicity of the strands (Table S3).

Table S3. Solvent gradients used in the HPLC purification of Cy3/Cy5 modified oligonucleotides.

Time/mins	0.1M TEAA in HPLC water/%	acetonitrile/%
0	95	5
15	76	24
20	57	43
25	57	43
25.1	0	100
35	0	100
35.1	95	5
41	95	5

Oligonucleotide characterisation

Samples showing >95% purity by analytical HPLC were deemed sufficiently pure for use in experiments. Samples showing <95% purity were repurified by semi preparative HPLC. The characterisation of pure oligonucleotide samples was performed by negative mode electrospray mass spectrometry. Sample concentrations were determined by optical density at 260nm using a BioSpec-nano micro-volume UV-Vis spectrophotometer (nanodrop) from Shimadzu and the Beer Lambert law, with extinction coefficients obtained from Integrated DNA Technologies' OligoAnalyzer.

Synthesis of fluorescent LOC probes

Adapted from Wilks[12]

DMF (100ml) was degassed for at least 30 minutes before use. 1mM stock solutions of bischolesterol dye azides and CuI.P(OEt$_3$) in degassed DMF (1ml) were prepared. Alkyne modified DNA (200µM) in Milli-Q water (1ml) stocks were also prepared.

For 3ml conjugation reactions in 4ml vials was added:

- 2.1ml degassed DMF
- 300µl of the assigned azide stock

- 300µl of CuI.P(OEt$_3$) stock
- 300µl of DNA alkyne stock

Reactions contained 20µM DNA alkyne, 100µM azide, and 100µM CuI.P(OEt$_3$). Argon was bubbled into the solution throughout additions and the vial was immediately sealed after the final addition. Vials were then left shaking overnight. Reactions were concentrated to 0.3ml on a speed vac and diluted to 1ml with Milli-Q water.

Purification of fluorescent LOC probes

The following HPLC method was used to purify the fluorescent LOC probes with a DNAPac RP 4µm 10 x 150mm prep column from Thermo Fisher. 1ml of sample was injected with a run time of 45 minutes for each sample, at a flow rate of 3ml/min. The column was heated to 60°C prior to sample injection. Samples were monitored at UV absorbances of 260nm for DNA, 550nm for Cy3 containing samples, and 645nm for Cy5 containing samples.

Table S4. Solvent gradients used in the purification of fluorescent LOC probes.

Time/mins	0.1M TEAA in HPLC water/%	acetonitrile/%
0	95	5
30	0	100
40	0	100
40.1	95	5
45	95	5

Collected fractions were evaporated to dryness, diluted to 1ml in Milli-Q water, and desalted using a NAP-10 column (GE Healthcare), whilst eluting to 1.5ml. Purity was determined by analytical HPLC using a DNAPac RP 4µm 2.1 x 100mm column. The column was heated to 60°C prior to sample injection. 70µl of sample was injected with a run time of 45 minutes for each sample, at a flow rate of 0.3ml/min. Solvent gradients used were identical to semi preparative HPLC (Table S4). The UV/vis absorbance of each run was monitored at 260nm, 550nm, and 645nm.

Fluorescent LOC probe characterisation

Samples showing >95% purity by analytical HPLC were deemed sufficiently pure for use in experiments. Samples showing <95% purity were repurified by semi preparative HPLC.

The characterisation of pure samples was performed by negative mode electrospray mass spectrometry. Sample concentrations were determined by optical density at 260nm using a BioSpec-nano micro-volume UV-Vis spectrophotometer (nanodrop) from Shimadzu and the Beer Lambert law.

Oligonucleotide and fluorescent LOC probe data

Table S5. Oligonucleotide and fluorescent LOC probe data.

Name	Sequence (5'→3')	Predicted mass (m/z)	Observed mass (m/z)
**TBA15 alkyne**	alkyne-GGT TGG TGT GGT TGG	4886	4886
**TBA29 alkyne**	alkyne-AGT CCG TGG TAG GGC AGG TTG GGG TGA CT	9246	9246
**cDNA**	AGT CAC CCC AAC CTC ACG TTT TCG TCC AAC CAC ACC AAC C	11989	11989
**scrambled DNA**	CGC CCC AAC CCG CCC ACC CAA GCG ACC CGT CCC CCG CTC AC	12227	12227
**cDNA to all of Cy5-bischol-TBA15**	TTT TTT TTT TTT TTT ACG TTT TCG TCC AAC CAC ACC AAC C	12048	12049
**cDNA to half of Cy3-bischol-TBA29**	AGT CAC CCC AAC CTC ACG TTT TCG TTT TTT TTT TTT TTT T	12070	12070
**Cy5-TBA15**	Cy5- GGT TGG TGT GGT TGG	5257	5256
**Cy3-TBA29**	Cy3- AGT CCG TGG TAG GGC AGG TTG GGG TGA CT	9589	9588
**Cy5-bischol-TBA15**	bischol Cy5- GGT TGG TGT GGT TGG	6254	6254
**Cy3-bischol-TBA29**	bischol Cy3- AGT CCG TGG TAG GGC AGG TTG GGG TGA CT	10585	10584

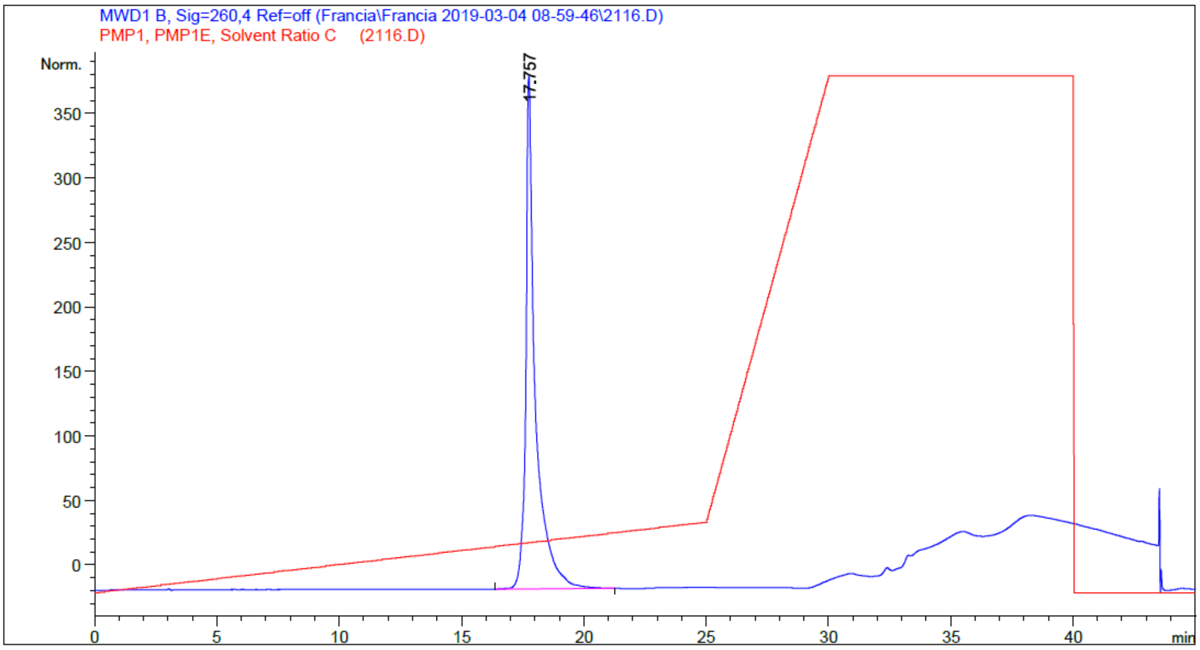

===

Area Percent Report

===

Sorted By : Signal
Multiplier : 1.0000
Dilution : 1.0000
Do not use Multiplier & Dilution Factor with ISTDs

Signal 1: MWD1 B, Sig=260,4 Ref=off

Peak #	RetTime [min]	Type	Width [min]	Area [mAU*s]	Height [mAU]	Area %
1	17.757	BB	0.3696	9822.98730	358.53177	100.0000

Figure S16. TBA15 alkyne HPLC chromatogram.

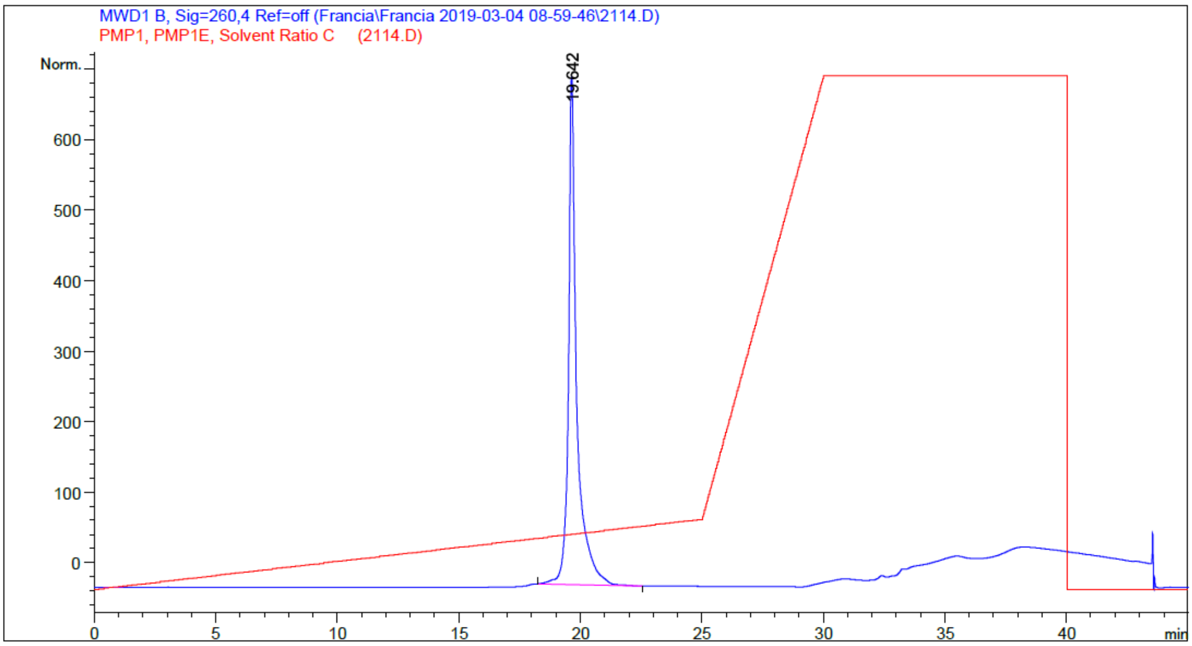


```
=====================================================================
                         Area Percent Report
=====================================================================

Sorted By             :      Signal
Multiplier            :      1.0000
Dilution              :      1.0000
Do not use Multiplier & Dilution Factor with ISTDs

Signal 1: MWD1 B, Sig=260,4 Ref=off

Peak RetTime Type  Width     Area      Height     Area
  #   [min]        [min]   [mAU*s]     [mAU]       %
----|-------|----|-------|----------|----------|--------|
   1  19.642 BB    0.3283 1.55263e4   650.77875 100.0000
```

Figure S17. TBA29 alkyne HPLC chromatogram.

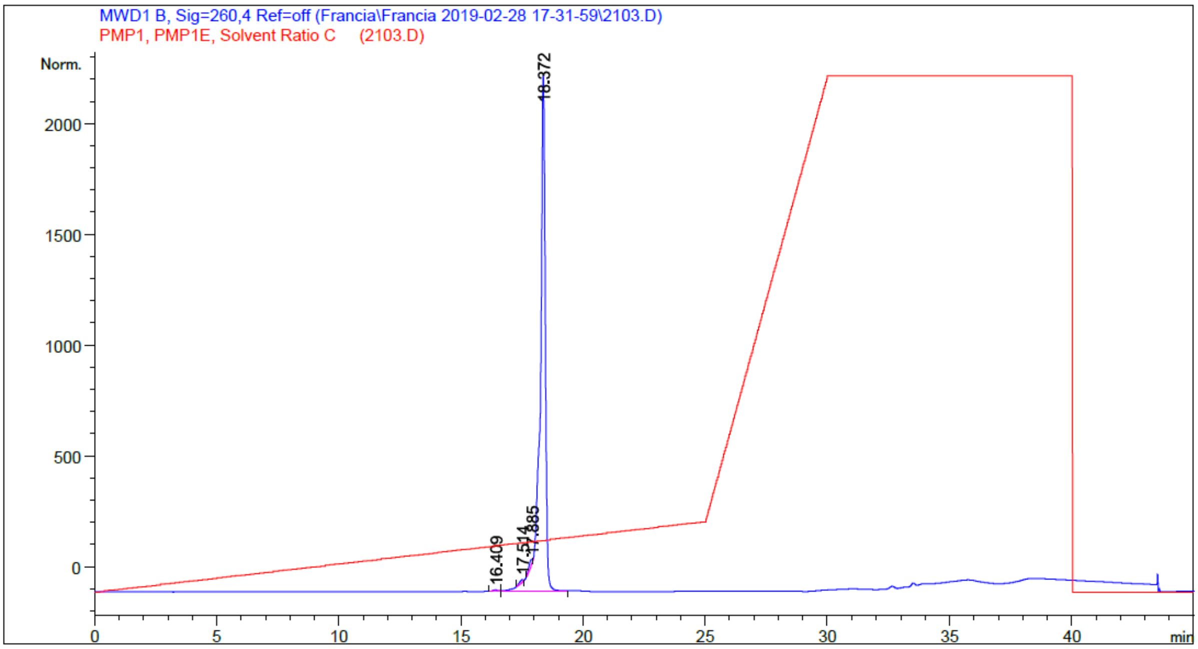


```
======================================================================
                          Area Percent Report
======================================================================

Sorted By             :      Signal
Multiplier            :      1.0000
Dilution              :      1.0000
Do not use Multiplier & Dilution Factor with ISTDs

Signal 1: MWD1 B, Sig=260,4 Ref=off

Peak RetTime Type  Width     Area      Height     Area
  #   [min]        [min]   [mAU*s]     [mAU]        %
----|-------|----|-------|----------|----------|--------|
1   16.409 BB    0.1499   48.70298    4.90080   0.1518
2   17.514 BV E  0.1795  132.26009   10.60700   0.4123
3   17.885 VV E  0.1717  271.02972   22.95517   0.8449
4   18.372 VB R  0.2139 3.16252e4  2097.33765  98.5909
```

Figure S18. cDNA HPLC chromatogram.

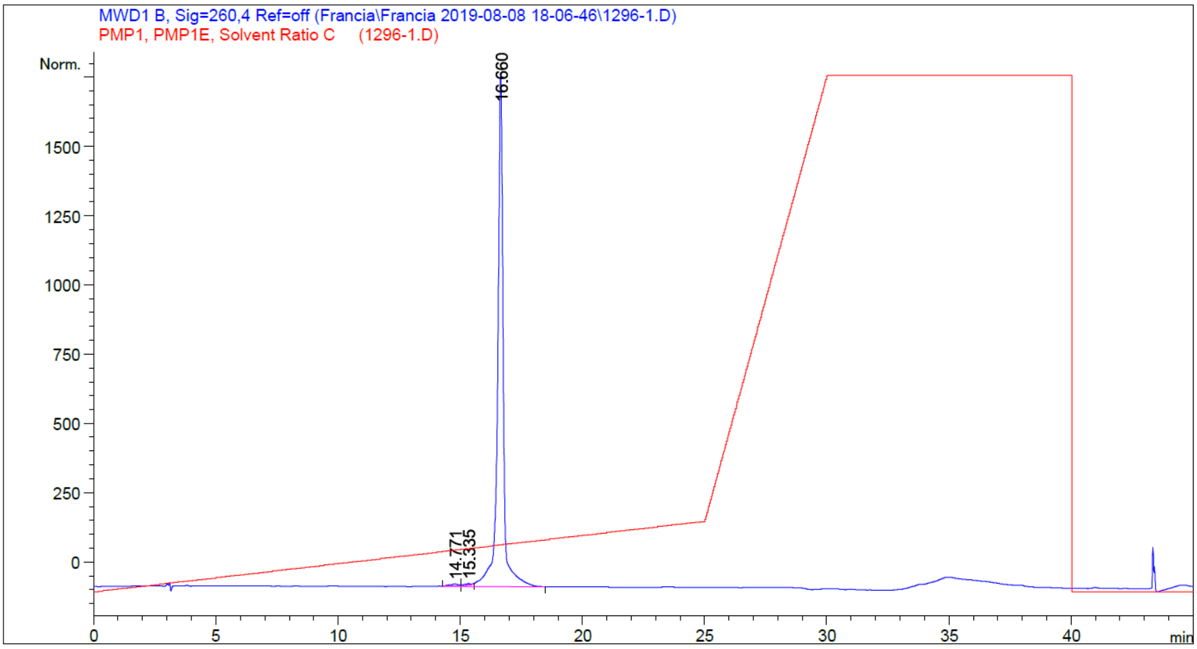


```
=====================================================================
                          Area Percent Report
=====================================================================

Sorted By             :      Signal
Multiplier            :      1.0000
Dilution              :      1.0000
Do not use Multiplier & Dilution Factor with ISTDs

Signal 1: MWD1 B, Sig=260,4 Ref=off

Peak RetTime Type  Width     Area      Height     Area
  #   [min]        [min]    [mAU*s]    [mAU]       %
----|-------|----|-------|----------|----------|--------|
  1  14.771 BV E  0.3352  190.22696    7.67266   0.6934
  2  15.335 VV E  0.2933  115.20855    5.56917   0.4200
  3  16.660 VB R  0.2363 2.71267e4  1662.82324  98.8866
```

Figure S19. Scrambled DNA HPLC chromatogram.

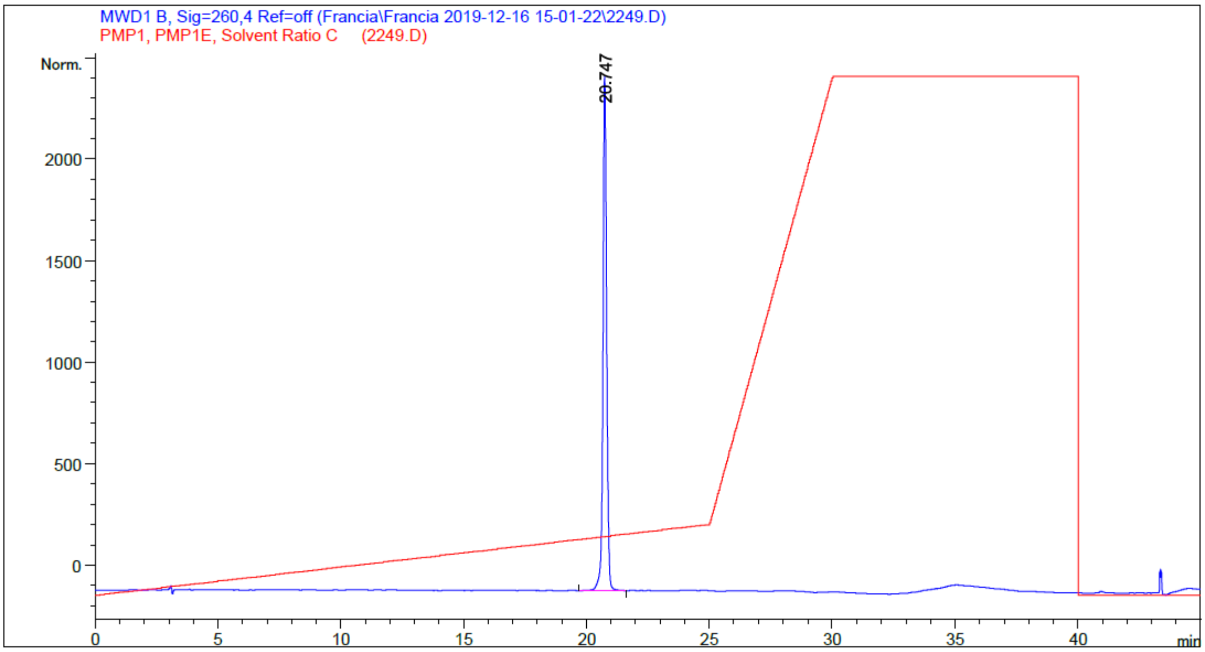


```
=====================================================================
                         Area Percent Report
=====================================================================

Sorted By             :      Signal
Multiplier            :      1.0000
Dilution              :      1.0000
Do not use Multiplier & Dilution Factor with ISTDs

Signal 1: MWD1 B, Sig=260,4 Ref=off

Peak RetTime Type  Width     Area      Height     Area
  #   [min]        [min]   [mAU*s]     [mAU]        %
----|-------|----|-------|----------|----------|--------|
   1  20.747 VB R  0.1620 2.42990e4  2286.69849 100.0000
```

Figure S20 cDNA to all of Cy5-bischol-TBA15 DNA HPLC chromatogram.

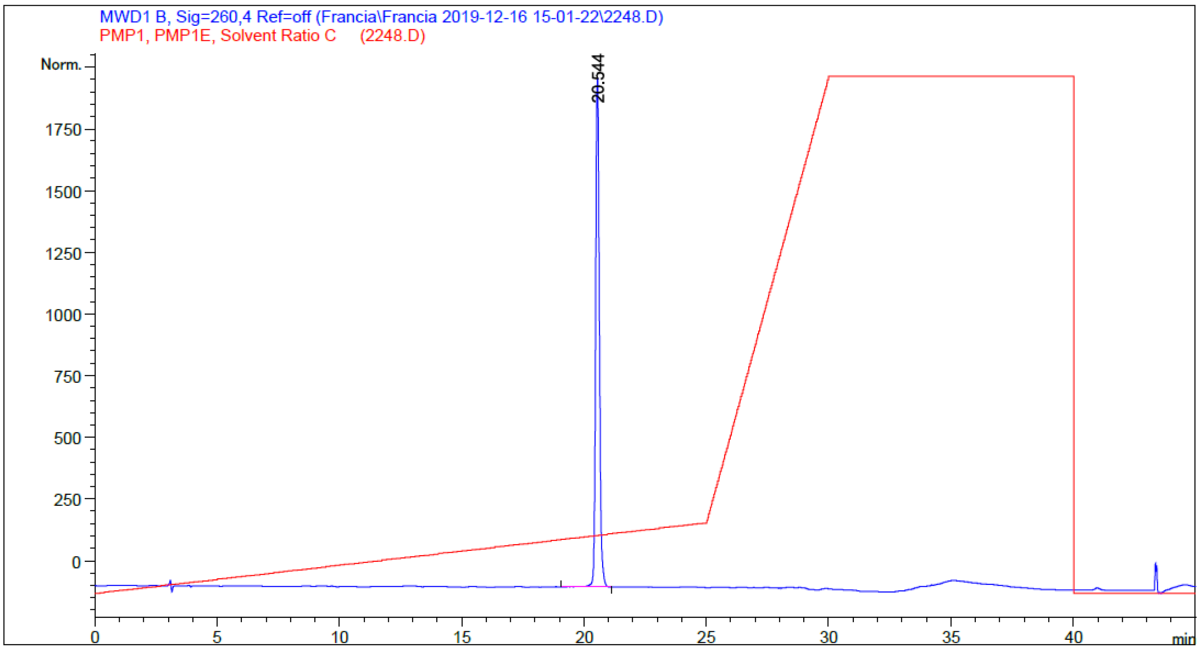


```
=====================================================================
                         Area Percent Report
=====================================================================

Sorted By             :      Signal
Multiplier            :      1.0000
Dilution              :      1.0000
Do not use Multiplier & Dilution Factor with ISTDs

Signal 1: MWD1 B, Sig=260,4 Ref=off

Peak RetTime Type  Width     Area      Height     Area
  #   [min]        [min]   [mAU*s]     [mAU]        %
----|-------|----|-------|----------|----------|--------|
   1  20.544 VB R  0.1608 1.96377e4  1865.41064 100.0000
```

Figure S21 cDNA to half of Cy3-bischol-TBA29 HPLC chromatogram.

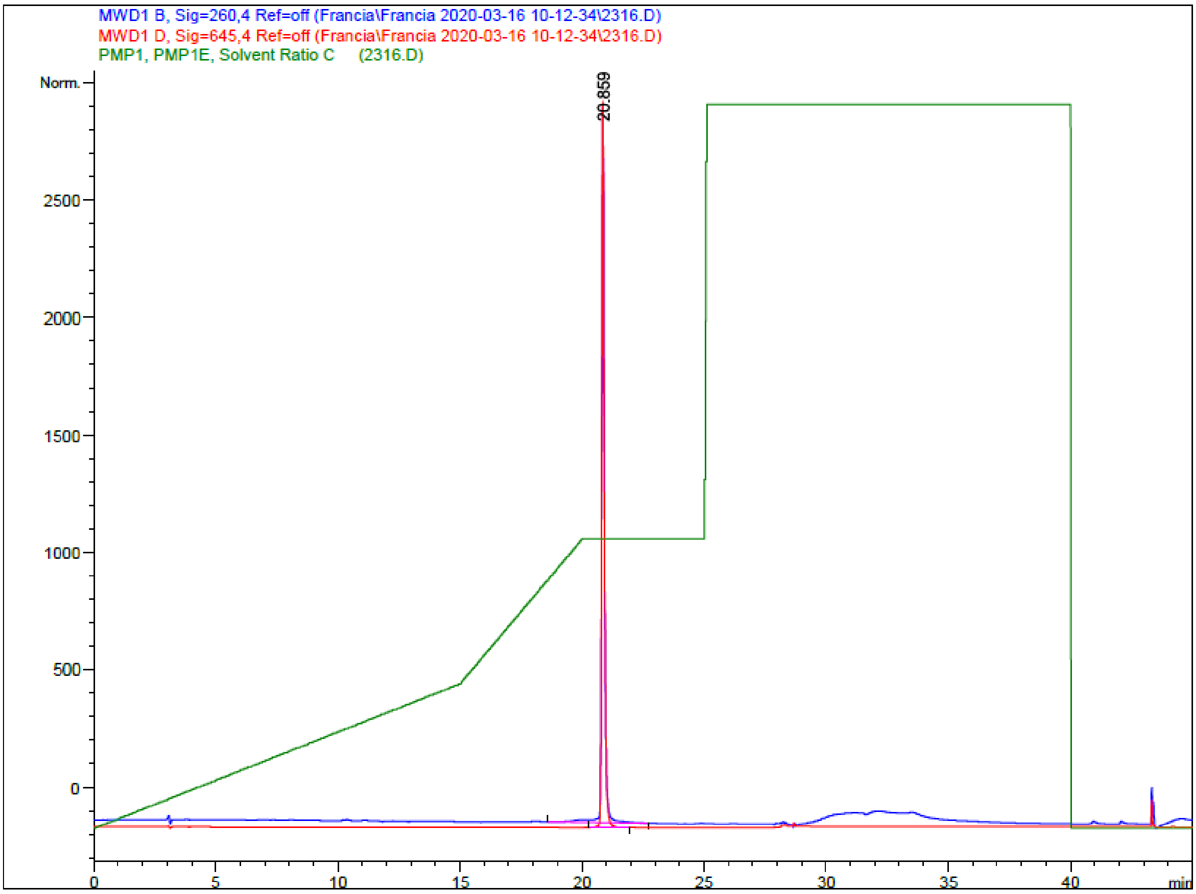


```
=====================================================================
                         Area Percent Report
=====================================================================

Sorted By             :      Signal
Multiplier            :      1.0000
Dilution              :      1.0000
Do not use Multiplier & Dilution Factor with ISTDs


Signal 1: MWD1 B, Sig=260,4 Ref=off

Peak RetTime Type  Width     Area      Height     Area
  #   [min]        [min]   [mAU*s]     [mAU]        %
----|-------|----|-------|----------|----------|--------|
   1  20.859 VV R  0.1093 1.59652e4  2159.71997 100.0000

Signal 2: MWD1 D, Sig=645,4 Ref=off

Peak RetTime Type  Width     Area      Height     Area
  #   [min]        [min]   [mAU*s]     [mAU]        %
----|-------|----|-------|----------|----------|--------|
   1  20.859 BV R  0.1101 2.03728e4  2760.38062 100.0000
```

Figure S22. Cy5-TBA15 HPLC chromatogram.

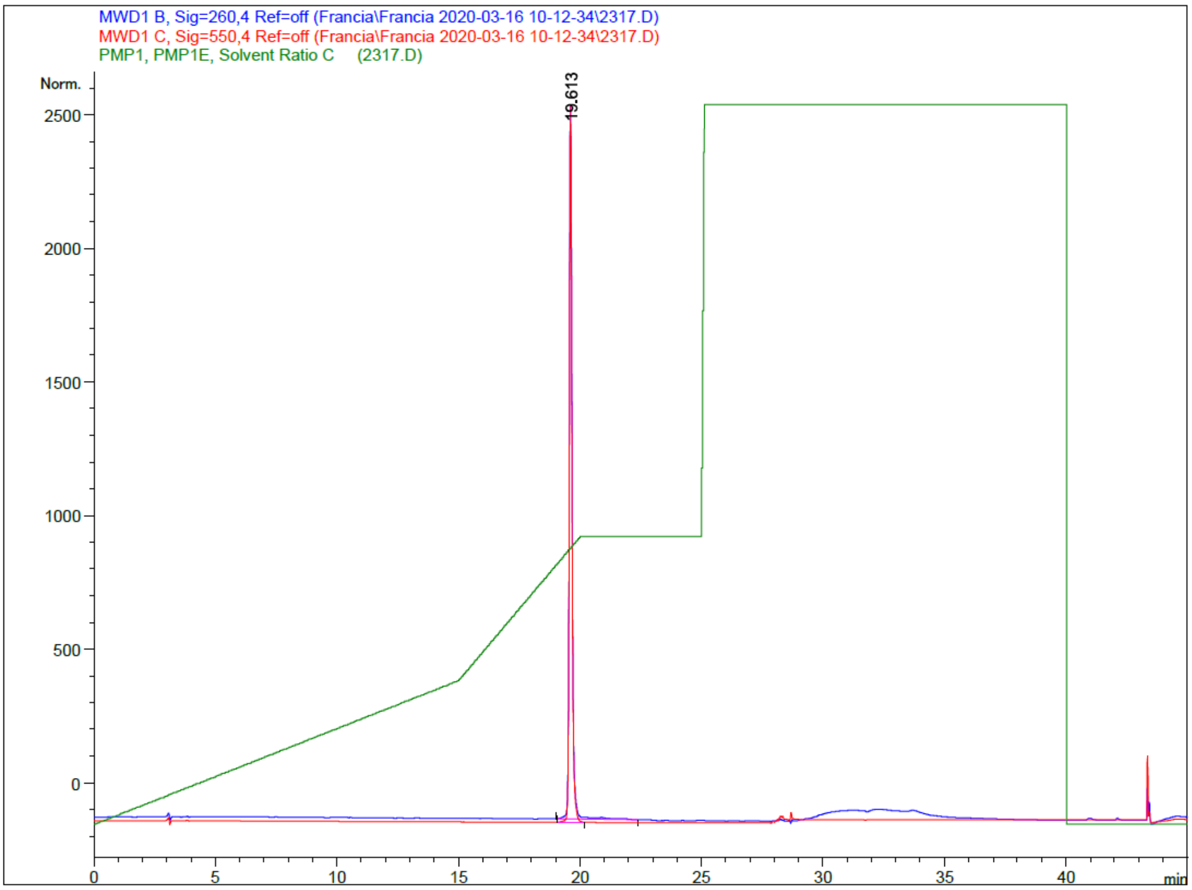


```
=====================================================================
                      Area Percent Report
=====================================================================

Sorted By              :      Signal
Multiplier             :      1.0000
Dilution               :      1.0000
Do not use Multiplier & Dilution Factor with ISTDs

Signal 1: MWD1 B, Sig=260,4 Ref=off

Peak RetTime Type  Width     Area      Height     Area
  #   [min]        [min]    [mAU*s]    [mAU]        %
----|-------|----|-------|----------|----------|--------|
   1  19.613 BV R  0.1280 2.03378e4  2411.36182 100.0000

Signal 2: MWD1 C, Sig=550,4 Ref=off

Peak RetTime Type  Width     Area      Height     Area
  #   [min]        [min]    [mAU*s]    [mAU]        %
----|-------|----|-------|----------|----------|--------|
   1  19.613 BB    0.1197 8988.95020 1144.17346 100.0000
```

Figure S23. Cy3-TBA29 HPLC chromatogram.

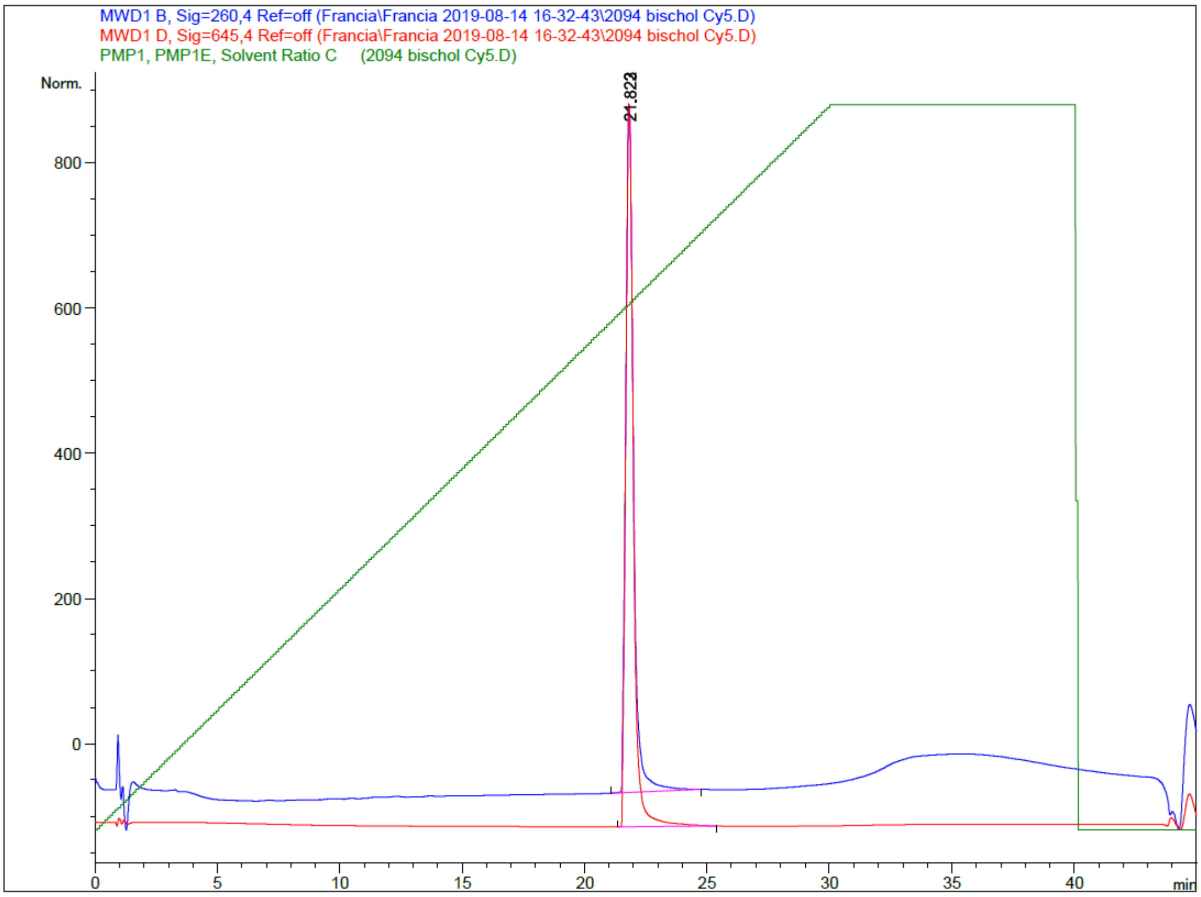

===

Area Percent Report

===

Sorted By : Signal
Multiplier : 1.0000
Dilution : 1.0000
Do not use Multiplier & Dilution Factor with ISTDs

Signal 1: MWD1 B, Sig=260,4 Ref=off

Peak #	RetTime [min]	Type	Width [min]	Area [mAU*s]	Height [mAU]	Area %
1	21.822	BB	0.3403	1.53547e4	688.16956	100.0000

Signal 2: MWD1 D, Sig=645,4 Ref=off

Peak #	RetTime [min]	Type	Width [min]	Area [mAU*s]	Height [mAU]	Area %
1	21.823	BB	0.3379	1.84627e4	835.30975	100.0000

Figure S24. Cy5-bischol-TBA15 HPLC chromatogram.

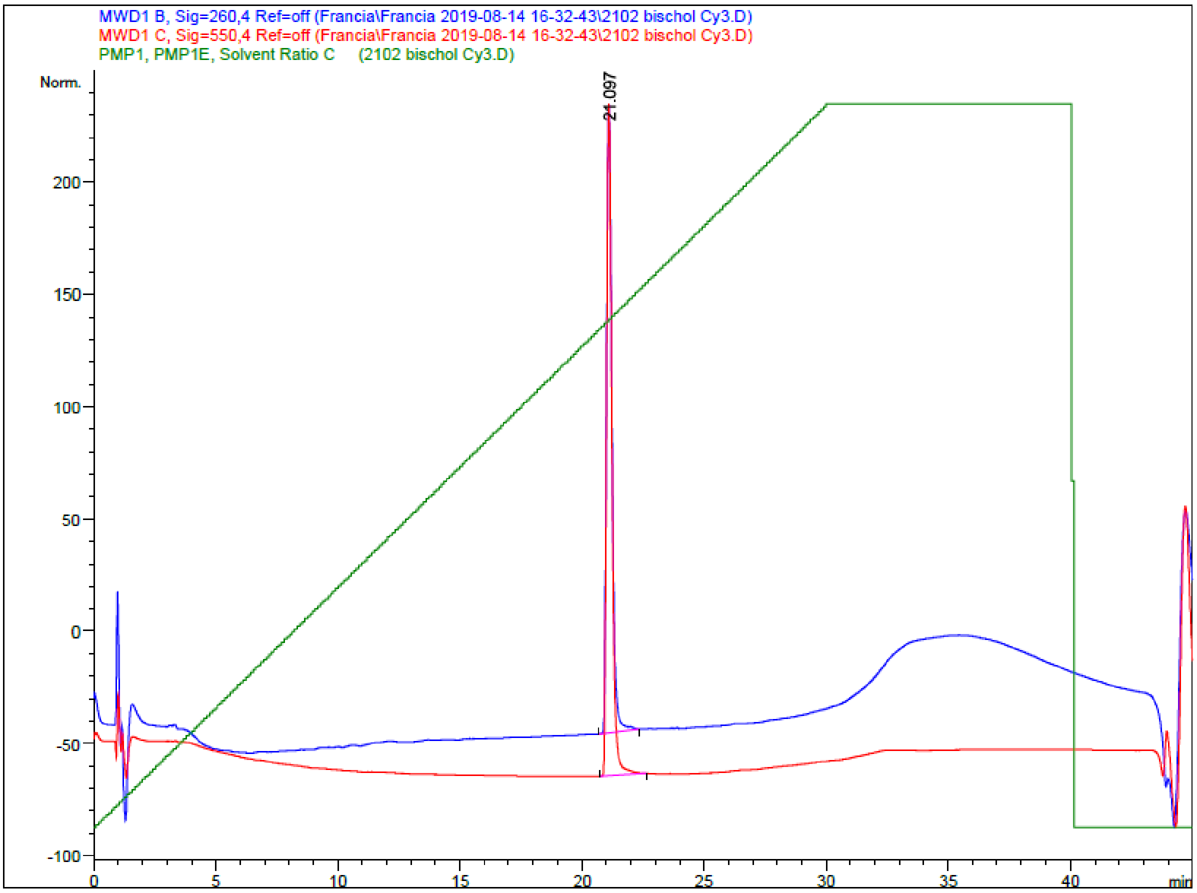


```
=====================================================================
                         Area Percent Report
=====================================================================

Sorted By             :      Signal
Multiplier            :      1.0000
Dilution              :      1.0000
Do not use Multiplier & Dilution Factor with ISTDs

Signal 1: MWD1 B, Sig=260,4 Ref=off

Peak RetTime Type  Width     Area      Height     Area
  #   [min]        [min]   [mAU*s]     [mAU]       %
----|-------|----|-------|----------|----------|--------|
   1  21.097 BV R  0.2299 3766.23706  252.60574 100.0000

Signal 2: MWD1 C, Sig=550,4 Ref=off

Peak RetTime Type  Width     Area      Height     Area
  #   [min]        [min]   [mAU*s]     [mAU]       %
----|-------|----|-------|----------|----------|--------|
   1  21.097 BB    0.2311 1488.08826   99.17192 100.0000
```

Figure S25. Cy3-bischol-TBA29 HPLC chromatogram.

Additional Data

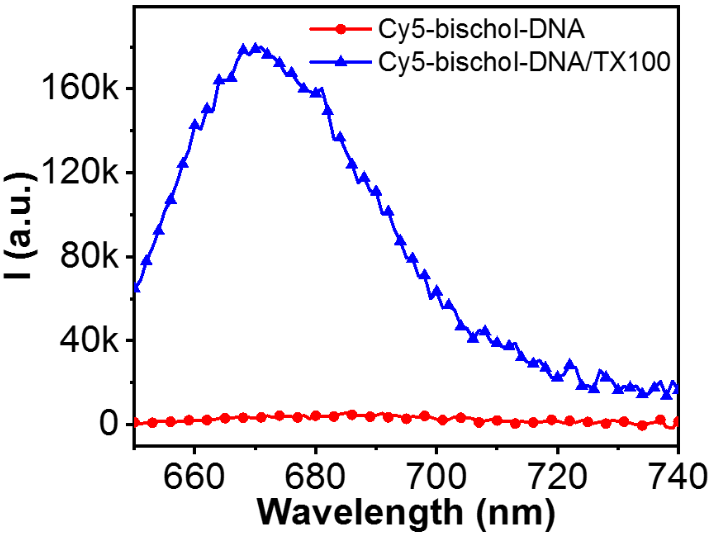

Figure S26. Representative fluorescence emission spectra Cy5-bischol-DNA/TX100 (blue triangle), and Cy5-bischol-DNA (red circle) excited at 622 nm. Concentrations were kept equal at 200 nM.

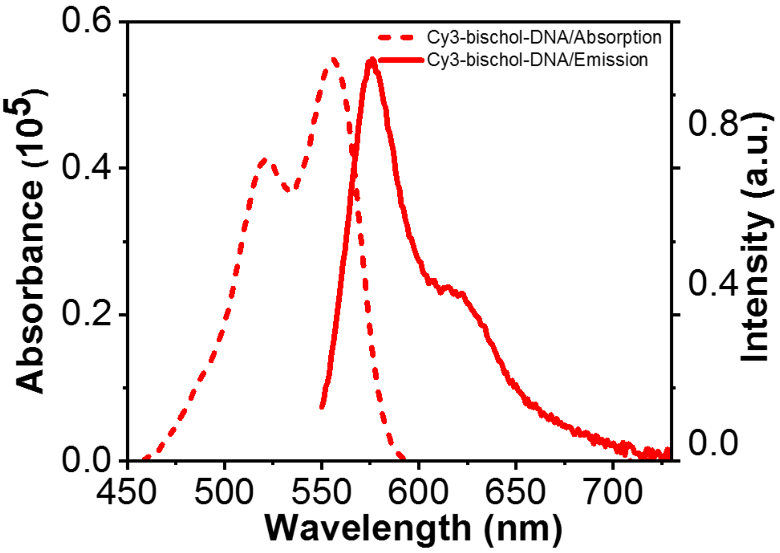

Figure S27. Absorption (dashed line) and emission spectra (solid line) for Cy3-bischol-DNA in 10 mM Tris buffer+150 mM NaCl. The samples were excited at 522 nm.

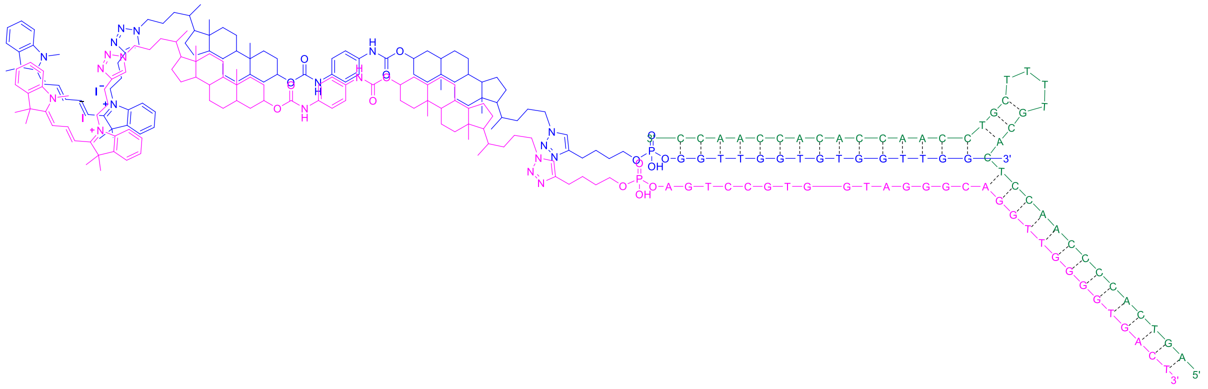

Figure S28. Assembly of the probes Cy5-bischol-TBA15 (in blue) and Cy3-bischol-TBA29 (in pink) in the presence of complementary DNA (in green) resulting in FRET.

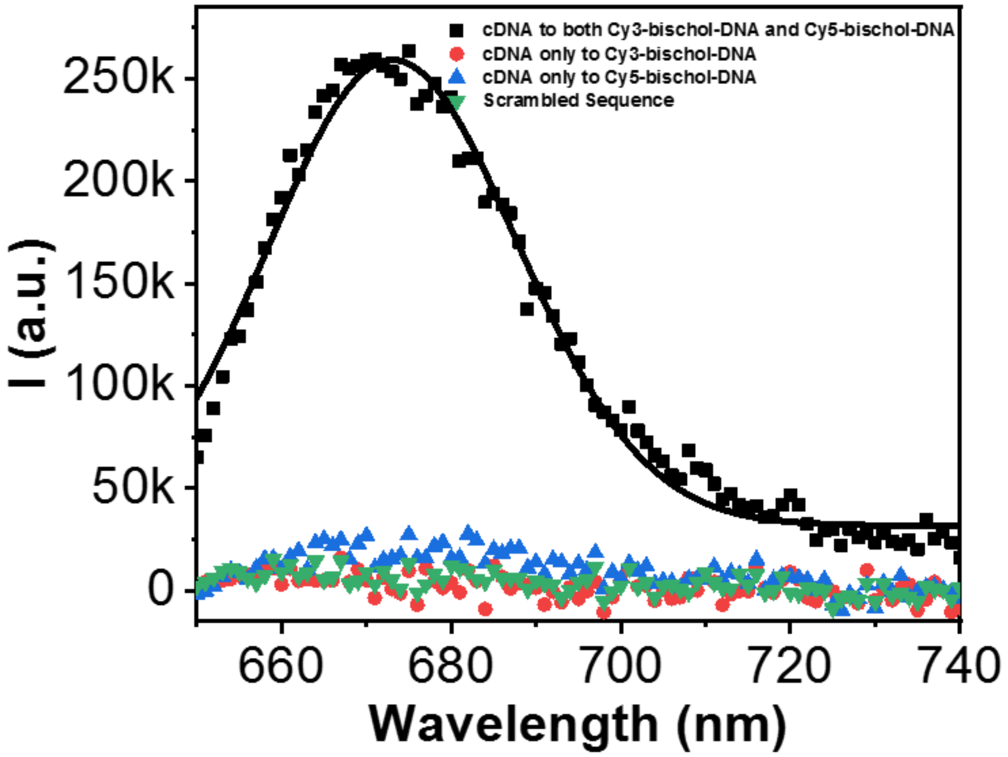

Figure S29. Background corrected Cy5 emission upon Cy3 excitation (522 nm) for dye-bischol-DNA/TX100 incubated with DNA strands complementary to both Cy3-bischol-DNA and Cy5-bischol-DNA (black square), only to the Cy3-bischol-DNA (red circle), the Cy5-bischol-DNA (blue triangle), or scrambled sequence (green triangle). DNA concentrations were kept equal at 100 nM.

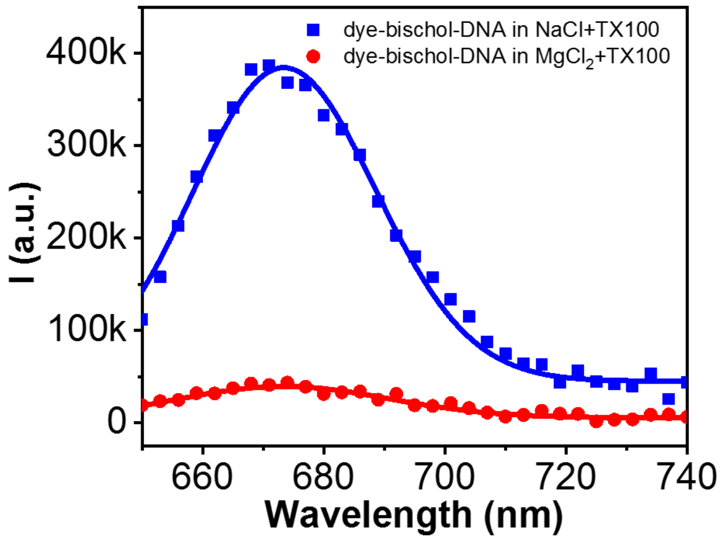

Figure S30. Background-corrected Cy5 emission of dye-bischol-DNA in 10 mM Tris buffer+150 mM NaCl+0.04% TX100 (blue square), and dye-bischol-DNA in 10 mM Tris buffer+150 mM $MgCl_2$+0.04% TX100 (red circle) after 30 min incubation with 200 nM cDNA. The samples were excited at 522 nm.

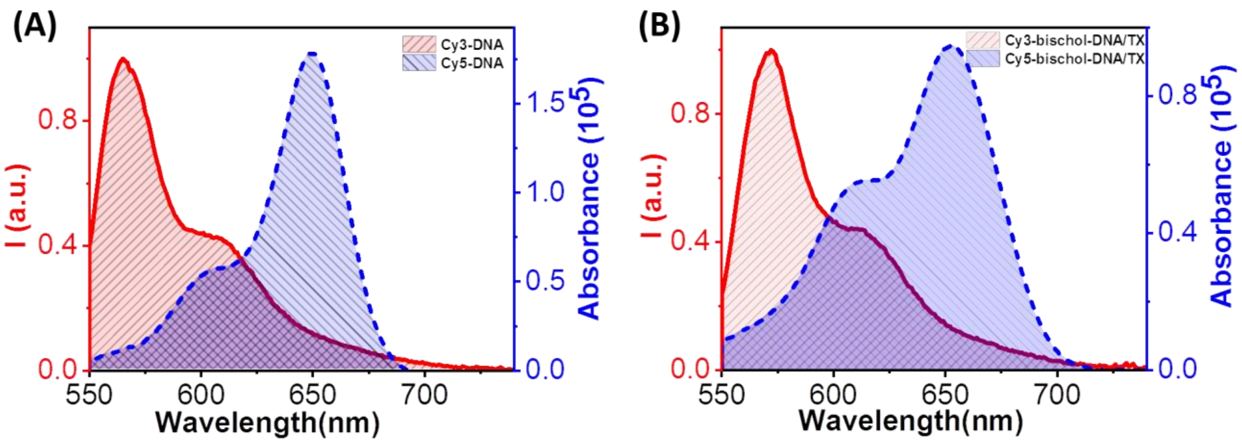

Figure S31. Spectral overlap of normalized emission spectrum of (A) Cy3-DNA (solid line, red) and UV/vis absorption (dashed line, blue) of Cy5-DNA and (B) Cy3-bischol-DNA/TX100 and Cy5-bischol-DNA/TX100.

Table S6. Spectral properties of Cy3 and Cy5 conjugated to DNA and bischol-DNA/TX100.

FRET Pair	Overlap Integral J ($nm^4/(M\ cm)$)	R_0 (nm)	Donor QY
Cy3-DNA/Cy5-DNA	2.8×10^{16}	6.61	0.15
Cy3-bischol-DNA/Cy5-bischol-DNA (TX100)	3.3×10^{16}	6.76	0.15

The spectral overlap integral (λ) for FRET pairs (Cy3-DNA/Cy5-DNA, Cy3-bischol-DNA/Cy5-bischol-DNA-TX100) is calculated using the absorption and emission spectra of dye-DNA and dye-bischol-DNA-TX100 as shown in Figure S29. quantum yield (QY) values are obtained from the reference literature.[8, 9] The corresponding Förster radius is calculated with the following formula:

$$R_0^6 = \frac{9000(\ln 10)K^2QY_D}{128\pi^5 N_{Av} n^4} \times J(\lambda)$$

Where k^2 (K^2=2/3) is the dipole orientation factor, QY_D is the fluorescence quantum yield of the donor molecule when the acceptor is absent, N_{AV} is Avogadro's number, *n* is the medium's refractive index (*n*=1.33) and J is the spectral overlap integral representing the overlap of donor's emission and acceptor's absorption spectra.

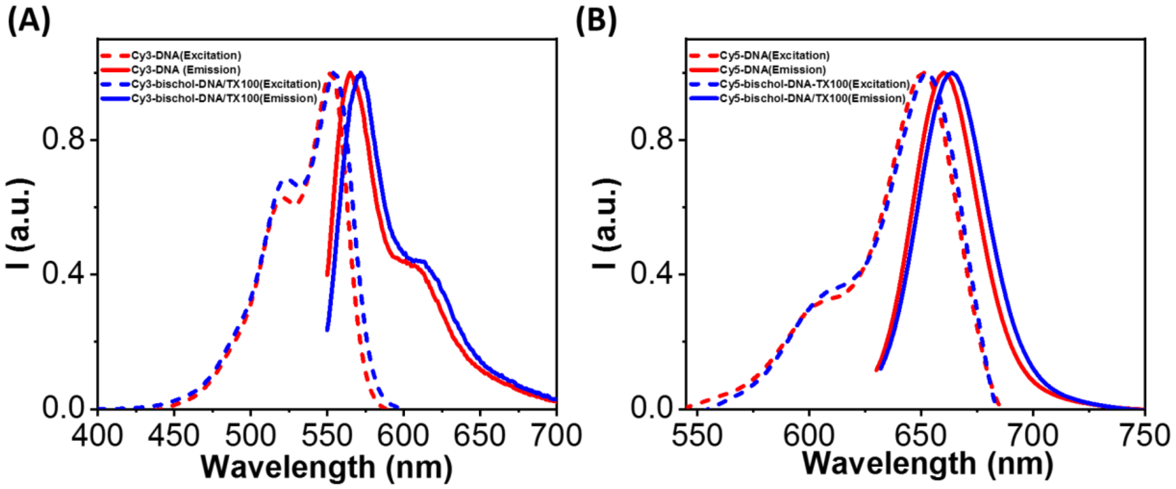

Figure S32. Excitation (dashed line) and emission spectra (solid line) for (A) Cy3-DNA (red)/Cy3-bischol-DNA/TX100 (blue) and (B) Cy5-DNA (red)/ Cy5-bischol-DNA/TX100 (blue). Cy3 and Cy5 were excited at 522 and 622 nm, respectively.

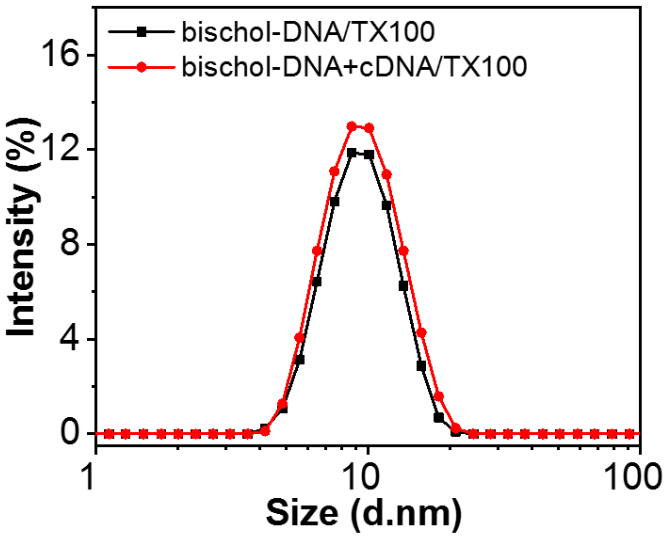

Figure S33. DLS data indicating the average size distribution of the mixture of 125 nM bischol-TBA29 and 200 nM bischol-TBA15 in 10 mM Tris+150 mM NaCl + 0.04% TX100 before (black square) and after (red circle) 30min incubation with 200nM cDNA.

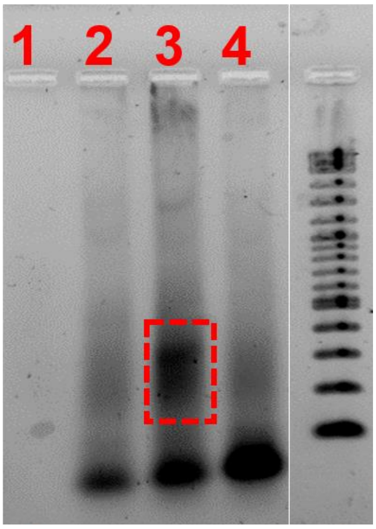

Figure S34. Gel analysis of 0.04% TX100 (lane 1), 125 nM Cy3-bischol-DNA/TX100 + 200 nM Cy5-bischol-DNA/TX100 (lane 2), 125 nM Cy3-bischol-DNA/TX100 + 200 nM Cy5-bischol-DNA/TX100 +100 nM cDNA (lane 3), and 125 nM Cy3-bischol-DNA/TX100 + 200 nM Cy5-bischol-DNA/TX100 + 100 nM scrambled sequence (lane 4). A DNA ladder is shown at the right (0.1-10kbp ladder).

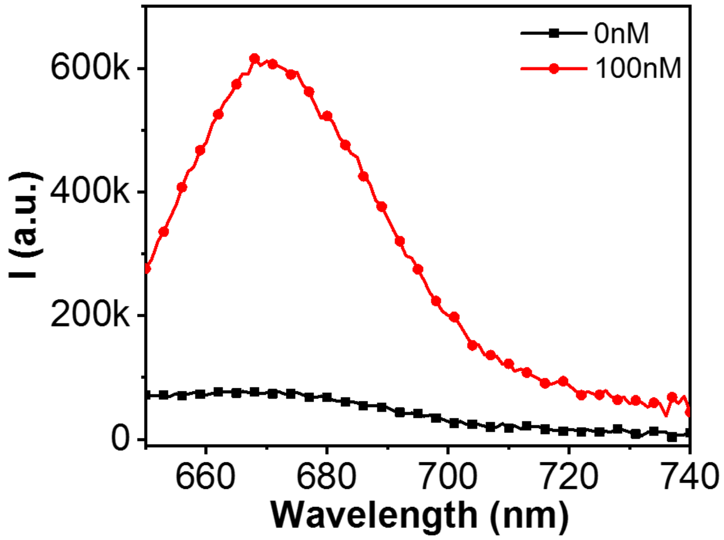

Figure S35. Cy5 emission of dye-bischol-DNA in 10 mM Tris buffer+150 mM NaCl+0.04% TX100 (black square) and dye-bischol-DNA in 10 mM Tris buffer+150 mM NaCl+0.04% TX100 +100 nM cDNA (red circle). The samples were excited at 522 nm.

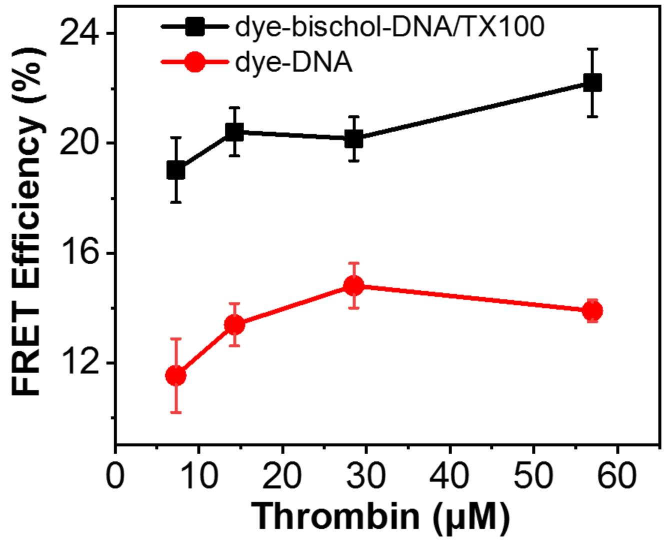

Figure S36. FRET efficiency (I_{Cy5} /I_{Cy5} + I_{Cy3}) of dye-bischol-DNA/TX100 mixed micelles and dye-DNA as a function of Thrombin concentration.

References

1. Gerowska, M.; Hall, L.; Richardson, J.; Shelbourne, M.; Brown, T., Efficient Reverse Click Labeling of Azide Oligonucleotides with Multiple Alkynyl Cy-Dyes Applied to the Synthesis of Hybeacon Probes for Genetic Analysis. *Tetrahedron* **2012,** *68* (3), 857-864.
2. Ma, L.; Melegari, M.; Colombini, M.; Davis, J. T., Large and Stable Transmembrane Pores from Guanosine−Bile Acid Conjugates. *J. Am. Chem. Soc.* **2008,** *130* (10), 2938-2939.
3. Kobuke, Y.; Nagatani, T., Transmembrane Ion Channels Constructed of Cholic Acid Derivatives. *J. Org. Chem.* **2001,** *66* (15), 5094-5101.
4. Sharma, R.; Majer, F.; Peta, V. K.; Wang, J.; Keaveney, R.; Kelleher, D.; Long, A.; Gilmer, J. F., Bile Acid Toxicity Structure–Activity Relationships: Correlations between Cell Viability and Lipophilicity in a Panel of New and Known Bile Acids Using an Oesophageal Cell Line (Het-1a). *Biorg. Med. Chem.* **2010,** *18* (18), 6886-6895.
5. Sheldrick, G. M., A Short History of Shelx. *Acta Crystallogr., Sect. A: Found. Crystallogr.* **2008,** *64* (Pt 1), 112-122.
6. Sheldrick, G. M., Crystal Structure Refinement with Shelxl. *Acta Crystallogr., Sect. C: Struct. Chem.* **2015,** *71* (Pt 1), 3-8.
7. Dolomanov, O. V.; Bourhis, L. J.; Gildea, R. J.; Howard, J. A. K.; Puschmann, H., Olex2: A Complete Structure Solution, Refinement and Analysis Program. *J. Appl. Crystallogr.* **2009,** *42* (2), 339-341.
8. Spiriti, J.; Binder, J. K.; Levitus, M.; Van Der Vaart, A., Cy3-DNA Stacking Interactions Strongly Depend on the Identity of the Terminal Basepair. *Biophys. J.* **2011,** *100* (4), 1049-1057.
9. Sanborn, M. E.; Connolly, B. K.; Gurunathan, K.; Levitus, M., Fluorescence Properties and Photophysics of the Sulfoindocyanine Cy3 Linked Covalently to DNA. *J. Phys. Chem. B* **2007,** *111* (37), 11064-11074.
